# Different niches for stem cells carrying the same oncogenic driver affect pathogenesis and therapy response in myeloproliferative neoplasms

**DOI:** 10.1038/s43018-023-00607-x

**Published:** 2023-08-07

**Authors:** Elodie Grockowiak, Claudia Korn, Justyna Rak, Veronika Lysenko, Adrien Hallou, Francesca M. Panvini, Matthew Williams, Claire Fielding, Zijian Fang, Eman Khatib-Massalha, Andrés García-García, Juan Li, Reema A. Khorshed, Sara González-Antón, E. Joanna Baxter, Anjali Kusumbe, Bridget S. Wilkins, Anna Green, Benjamin D. Simons, Claire N. Harrison, Anthony R. Green, Cristina Lo Celso, Alexandre P. A. Theocharides, Simón Méndez-Ferrer

**Affiliations:** 1 National Health Service Blood and Transplant, Cambridge, UK; 2 Department of Haematology, University of Cambridge, Cambridge, UK; 3 Wellcome Trust-Medical Research Council Cambridge Stem Cell Institute, Cambridge, UK; 4 Department of Medical Oncology and Hematology, University of Zurich and University Hospital Zurich, Zurich, Switzerland; 5 Wellcome Trust-CRUK Gurdon Institute, University of Cambridge, Cambridge, UK; 6 Cavendish Laboratory, Department of Physics, University of Cambridge, Cambridge, UK; 7 Department of Applied Mathematics and Theoretical Physics, Centre for Mathematical Sciences, University of Cambridge, Cambridge, UK; 8 Department of Life Sciences, Sir Alexander Fleming Building, Imperial College London, London, UK; 9 The Sir Francis Crick Institute, London, UK; 10 The Kennedy Institute of Rheumatology, University of Oxford, Oxford, UK; 11 Guy’s and Saint Thomas’ NHS Foundation Trust, London, UK

**Keywords:** Myeloproliferative disease, Haematopoietic stem cells, Cancer

## Abstract

Aging facilitates the expansion of hematopoietic stem cells (HSCs) carrying clonal hematopoiesis-related somatic mutations and the development of myeloid malignancies, such as myeloproliferative neoplasms (MPNs). While cooperating mutations can cause transformation, it is unclear whether distinct bone marrow (BM) HSC-niches can influence the growth and therapy response of HSCs carrying the same oncogenic driver. Here we found different BM niches for HSCs in MPN subtypes. JAK–STAT signaling differentially regulates CDC42-dependent HSC polarity, niche interaction and mutant cell expansion. Asymmetric HSC distribution causes differential BM niche remodeling: sinusoidal dilation in polycythemia vera and endosteal niche expansion in essential thrombocythemia. MPN development accelerates in a prematurely aged BM microenvironment, suggesting that the specialized niche can modulate mutant cell expansion. Finally, dissimilar HSC-niche interactions underpin variable clinical response to JAK inhibitor. Therefore, HSC-niche interactions influence the expansion rate and therapy response of cells carrying the same clonal hematopoiesis oncogenic driver.

## Abstract (web summary)

Grockowiak et al. explore bone marrow niche heterogeneity in myeloproliferative neoplasms, polycytemia vera and essential thrombocytemia and find *JAK2*-mutated hematopoietic stem cells occupying distinct niches affecting cell growth and therapy response.

## Main

Somatic mutation-driven clonal hematopoiesis (CH) commonly arises with aging and is associated with increased risk of myeloid malignancies^1^. While CH-related mutations might provide hematopoietic stem cells (HSCs) with a competitive fitness advantage, it is unclear why small clones often remain indolent in the bone marrow (BM) for many years^1^. The most commonly acquired mutations affect the genes encoding DNA methyltransferase 3 alpha (*DNMT3A*), tet methylcytosine dioxygenase 2 (*TET2*), additional sex combs like 1 (*ASXL1*), Janus kinase 2 (*JAK2*) and tumor protein p53 (*TP53*)^1^. Among *JAK2* mutations in CH, *JAK*2^V617F^ is the most common and can lead to uncontrolled expansion of HSCs and erythroid, megakaryocytic and myeloid progenitors^1–5^. In myeloproliferative neoplasms (MPNs), *JAK2*^V617F^ is present in most cases with polycythemia vera (PV) and more than 50% of cases with essential thrombocytemia (ET)^2–5^. Moreover, nearly all remaining cases with ET exhibit mutations in the gene encoding calreticulin (*CALR*)^6,7^ but still depend on oncogenic JAK2–STAT signaling^8–10^. However, it is unclear how the same mutation or oncogenic pathway can give rise to different diseases with variable progression (with PV showing a higher transformation risk into secondary myelofibrosis or leukemia, compared with ET)^11,12^. Furthermore, JAK inhibitors, like ruxolitinib, are superior to second-line treatments in PV but not in ET^13–15^; however, the underlying reasons for this discrepancy are unknown. We hypothesized that the HSC-niche might impact the variable pathogenesis and therapy response observed in different MPN subtypes.

Previous studies suggested that niches near the bone surface (endosteal) promote HSC quiescence under stress^16–23^, while activated HSCs traffic in and out of the BM through sinusoids located further away from the bone surface^24^. These studies raise the possibility that different BM niches and their alterations during aging might influence the expansion of clones carrying CH-related somatic mutations in chronic inflammatory diseases, such as MPNs^25^. Supporting this possibility, endosteal HSC-niches are reduced in mice during aging^26–29^. In contrast, we found expanded central BM niches driving myeloid and megakaryocyte expansion during aging^28^. Therefore, we investigated whether the heterogeneity of BM HSC-niches might explain the different expansion of HSCs carrying the same CH driver mutation in ET and PV, and their overall response to JAK inhibitor.

## Results

### Different BM niches for human hematopoietic stem and progenitor cells in MPN subtypes

**Fig. 1:**
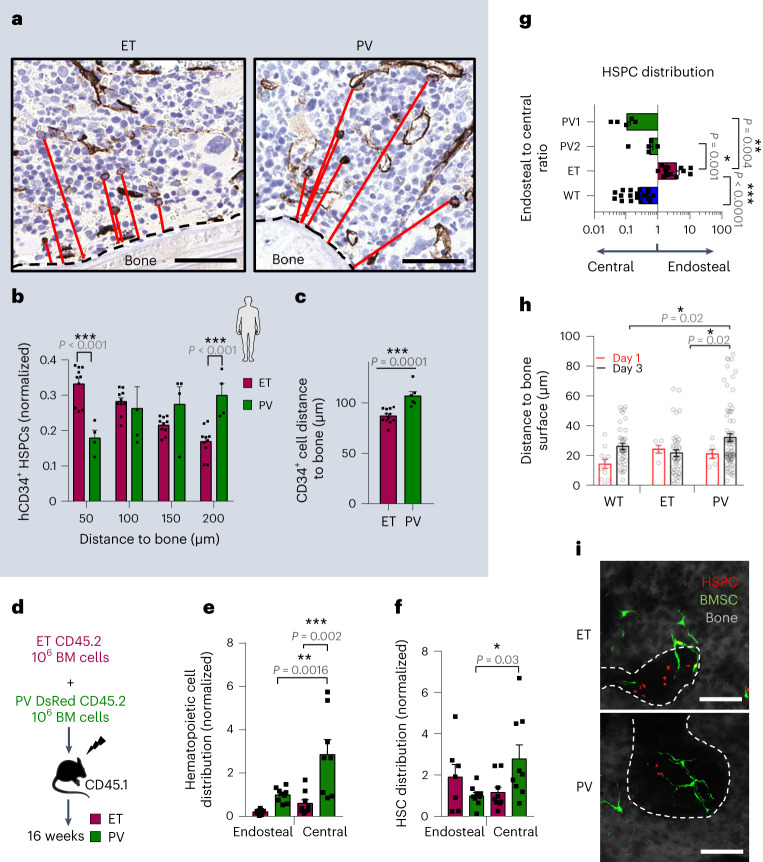
Different niches for HSPCs carrying the same oncogenic driver in MPN subtypes. **a**, Immunohistochemistry for CD34 and hematoxylin showing CD34^+^ hHSPCs and their shortest distance to the bone surface (red line) in BM sections from *JAK2*^V617F^-mutant human ET or PV. Scale bar, 30 µm. **b**, hHSPC frequency at distance ranges (in micrometers) from bone surface in ET (*n* = 12 patients) or PV (*n* = 6 patients). **c**, Mean distance between hHSPCs and the bone surface in ET (*n* = 12 patients) or PV (*n* = 6 patients). **d**–**f**, Competitive transplant with ET-like and PV-like BM cells. **d**, Outline of competitive transplantation: BM cells from CD45.2 ET-like mouse and DsRed PV-like mouse were transplanted together in irradiated CD45.1 recipients. **e**,**f**, BM distribution of ET-like or PV-like hematopoietic cells (CD45^+^) (**e**) and Lin^−^Sca1^+^cKit^+^ (LSK) mHSPCs (**f**) in recipient mice (*n* = 9 mice). **g**, BM distribution (endosteal:central ratio) of WT, PV-like or ET-like mHSPCs after noncompetitive transplantation of BM cells. PV1, *Scl-Cre*^ERT2^*;JAK2*^V617F^, *n* = 5 mice. PV2, *Mx1-Cre:JAK2*^V617F^, *n* = 5 mice. ET, *iVav-Cre;JAK2*^V617F^, *n* = 14 mice. WT, *n* = 18 mice. **h**, Distance to bone surface measured longitudinally through intravital imaging 1–3 days after transplantation of WT, ET-like or PV-like DsRed HSCs (*n* = 6 independent experiments). HSPCs at day 1, WT (*n* = 11 cells), ET (*n* = 5 cells), PV (*n* = 6 cells). HSPCs at day 3, WT (*n* = 40 cells), ET (*n* = 45 cells), PV (*n* = 74 cells). **i**, Representative BM Z-stacks 3 days after transplantation into *Nes-GFP* mice (with genetically labeled HSC-niche-forming cells^32^). Bone surface is depicted with a dashed line. Scale bar, 100 µm. *n* = 6 independent experiments. In **b**,**c** a two-sided Student’s *t*-test was used. In **e**–**h** a two-sided one-way analysis of variance (ANOVA) was used. In **b**,**c**,**e**–**g** each square dot is an organism. In **h** each dot is a cell. Data are shown as the mean ± s.e.m. *P < 0.05; **P < 0.01; ***P < 0.001. Source data

To investigate the BM distribution of human hematopoietic stem and progenitor cells (hHSPCs) in MPNs, CD34 immunohistochemistry was performed in BM biopsies (trephines) from diagnosed patients with ET or PV with a similar tumor size (allele burden). Interestingly, double the frequency of hHSPCs was found within 50 µm from the bone surface in ET, while twice as many hHSPCs were far (150–200 µm) from bone in PV (Fig. 1a,b). On average, hHSPCs were significantly closer to the bone surface in ET than in PV (Fig. 1c). These results suggested the possibility that hHSPCs carrying the same driver mutation exhibit different niche preferences associated with distinct MPN progression, which was subsequently investigated in mouse models.

### Distinct niche preferences in ET and PV

**Extended Data Fig. 1:**
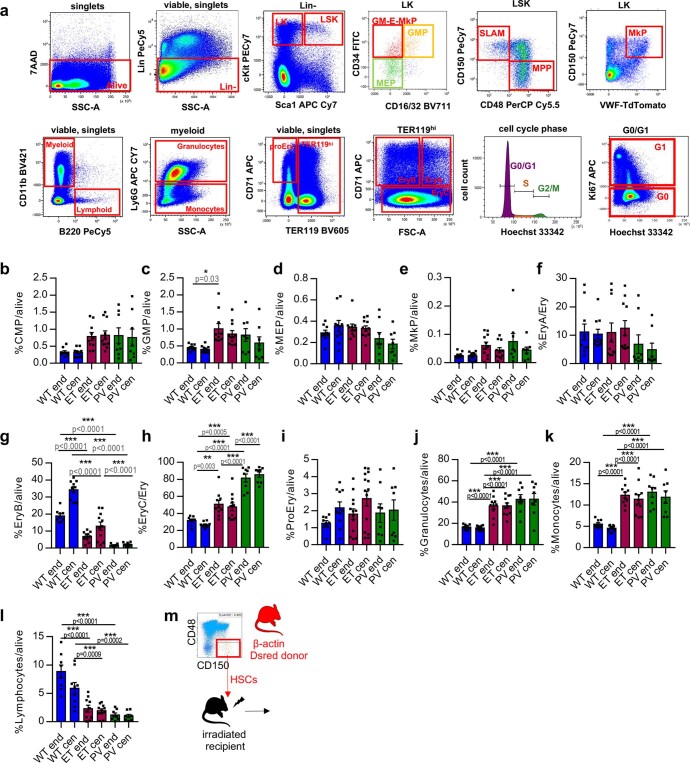
Different niches for HSPCs, but not their downstream progeny, in MPN subtypes. **a**, Gating strategy for the mouse hematopoietic populations. Lin^−^Sca1^+^cKit^+^ (LSK) hematopoietic stem and progenitor cells (HSPCs), LSK CD150^+^CD48^−^ hematopoietic stem cells (HSCs), LSK CD150^−^CD48^+^ multipotent progenitors (MPPs), Lin^−^cKit^+^Sca1^−^ CD34^+^CD16/32^−^Granulocyte/macrophage/megakaryocyte /erythrocyte progenitor (CMP), Lin^−^cKit^+^Sca1^−^CD34^+^CD16/32^+^ granulocyte-monocyte progenitors (GMP), Lin^−^cKit^+^ Sca1^−^CD34^−^CD16/32^−^ megakaryocyte-erythroid progenitors (MEP), Lin^−^ckit^−^Sca1^−^CD150^+^VWF^+^ megakaryocyte progenitors (MkP), B220^−^CD11b^+^Ly6G^+^ granulocytes, B220^−^CD11b^+^Ly6G^−^ monocytes, CD71^+^TER119^−^ pro-erythrocytes (ProEry), TER119^hi^CD71^+^FSC-A^hi^ EryA erythroblasts (EryA), TER119^hi^CD71^+^FSC-A^lo^ EryB erythroblasts (EryB), TER119^hi^CD71^−^ EryC erythroblasts (EryC), B220^+^ B lymphocytes (LYM). **b**-**l**, Frequencies of the hematopoietic populations gated on (**a**) in the endosteal (end) or central (cen) BM of WT mice transplanted with WT, ET-like or PV-like BM cells, 16w after transplantation (n=8-10 mice). **b**, Granulocyte/macrophage/megakaryocyte/erythrocyte progenitor (GM-E-MkP). **c**, Granulocyte-monocyte progenitors (GMP). **d**, Megakaryocyte-erythroid progenitors (MEP). **e**, Megakaryocyte progenitors (MkP). **f**, EryA erythroblasts (EryA). **g**, EryB erythroblasts (EryB). **h**, EryC erythroblasts (EryC). **i**, Pro-erythrocytes (ProEry). **j**, Granulocytes. **k**, Monocytes. **l**, Lymphocytes. **b**-**l**, Data are means±SEM. Each square dot is a mouse. Two-sided One-way ANOVA, *p<0.05; **p<0.01; ***p<0.001. **m**, Outline of intravital imaging. Labelled mHSCs were injected into *Nes-GFP* mice (with genetically labelled HSC niche-forming cells45) previously irradiated, to achieve sufficient BM homing, and visualized 1 and 3 days later through combined confocal and 2-photon microscopy. Source data

The hMPN was modeled in mice carrying the *JAK2*^V617F^ mutation driven by *Mx1-Cre* or *Vav1-Cre*, which respectively develop PV-like or ET-like MPNs^30^. *Mx1-Cre;JAK2*^V617F^ mice were intercrossed with β-actin-DsRed reporter mice to label PV-like hematopoietic cells. To stringently test the niche preferences of ET-like and PV-like hematopoietic cells, 10^6^ BM mononuclear cells from mice with phenotypic ET or PV were transplanted together into lethally irradiated congenic recipient mice (Fig. 1d). Sixteen weeks after transplantation, donor-derived hematopoietic chimerism was fivefold higher for PV-derived cells compared with ET-derived cells (Fig. 1e), reproducing the clinical observation that PV is more aggressive than ET^11,12^. Mouse HSPCs (mHSPCs) tended to distribute asymmetrically in the same bones, with ET-like and PV-like mHSPCs preferentially expanding in the endosteal or central BM, respectively (Fig. 1f). The divergent anatomical niche preference of PV-like or ET-like mHSPCs was confirmed in noncompetitive transplantations and using a PV model driven by the *Scl-Cre*^ERT2^ promoter^31^ (PV1; Fig. 1g) and affected mHSPCs, but not more mature progenitors (Extended Data Fig. 1a–l). These results suggest that HSCs carrying the same oncogenic driver (*JAK2*^V617F^), but causing different disease progression, expand in distinct BM niches.

To track mHSCs and their progeny in vivo, we isolated mHSCs from ET-like or PV-like mice intercrossed with β-actin-DsRed reporter mice. Labeled mHSCs were injected into *Nes-GFP* mice (with genetically labeled HSC-niche-forming cells^32^), which were lethally irradiated to achieve sufficient BM homing (Extended Data Fig. 1m). The interaction of mHSCs with their native niches was longitudinally studied through combined real-time two-photon and confocal live imaging within 24 h (before the first cell division) and after 3 days, following their proliferation and commitment yielding more HSCs and progenitors (HSPCs). Resembling wild-type (WT) mHSCs^16^, MPN mHSPCs homed near *Nes-GFP*^+^ cells; however, ET-like mHSPCs remained comparatively closer to the bone surface, while PV-like mHSPCs progressively moved away from the bone surface (Fig. 1h,i), which is indicative of distinct HSC-niche interactions in MPN subtypes over time.

### Differential BM niche remodeling in MPN subtypes

**Fig. 2:**
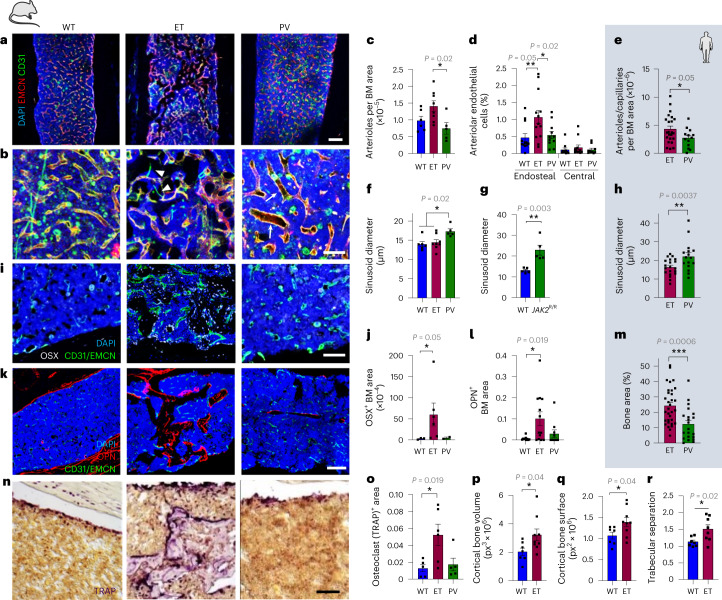
Asymmetric HSC-niche interactions cause differential vascular and stromal remodeling in MPN subtypes. **a**–**d**,**f**,**g**,**i**–**l**,**n**–**r**, Analysis of WT mice transplanted with WT (*n* = 10 mice), ET-like (*n* = 12 mice) or PV-like (*n* = 10 mice) BM cells (white background). **e**,**h**,**m**, Analysis of patients with MPN BM trephines (blue background). **a**,**b**, Immunofluorescence of CD31^+^ (green) or endomucin (EMCN)^+^ (red) blood vessels, representative image in **a** and quantification in **b**. Nuclei were counterstained with 4′,6-diamidino-2-phenylindole (DAPI) (blue). **c**, Arterioles (small caliper CD31^high^EMNC^lo^ vessels) per BM area. WT (*n* = 6 mice), ET (*n* = 8 mice), PV (*n* = 5 mice). **d**, Frequency of Sca1^hi^CD31^hi^ arteriolar endothelial cells among CD45^−^Ter119^−^ cells. WT (*n* = 12 mice), ET (*n* = 13 mice), PV (*n* = 11 mice). **e**, Arterioles or capillaries per BM area in patients’ trephines. ET (*n* = 23 patients), PV (*n* = 13 patients). **f**,**g**, Sinusoid diameter in transgenic (**f**) or knockin (**g**) models of MPN (compared with WT mice). **f**, WT (*n* = 6 mice), ET (*n* = 8 mice), PV (*n* = 5 mice). **g**, WT (*n* = 5 mice), *JAK2*^R/R^ (*n* = 5 mice). **h**, Sinusoid diameter in BM trephines from ET (*n* = 20 patients) or PV (*n* = 16 patients). **i**–**l**, Immunofluorescence (**i**,**k**) and quantification (**j**,**l**) of OSX^+^ osteoprogenitors (**i**, white) and OPN^+^ osteoblasts (**k**, red). Scale bar in **a** and **k**, 200 µm. **j**, WT (*n* = 3 mice), ET (*n* = 6 mice), PV (*n* = 2 mice). **l**, WT (*n* = 10 mice), ET (*n* = 13 mice), PV (*n* = 10 mice). **m**, Human BM trephine area occupied by bone (in percent). ET (*n* = 32 patients), PV (*n* = 21 patients). **n**,**o**, Representative staining (**n**) and quantification (**o**) of tartrate-resistant acid phosphatase (TRAP)^+^ osteoclast area. **o**, WT (*n* = 5 mice), ET (*n* = 6 mice), PV (*n* = 5 mice). Scale bar in **b**, **l** and **n**, 100 µm. **p**–**r**, Bone histomorphometry (µCT) analysis of WT (*n* = 7 mice) and ET-like (*n* = 9 mice) mice. **p**, Cortical bone volume. **q**, Cortical bone surface. **r**, Trabecular separation. Each square dot represents a mouse or individual. Data are shown as the mean ± s.e.m. *P < 0.05; **P < 0.01; ***P < 0.001. In **c**,**d**,**f**,**j**,**l** a two-sided, one-way ANOVA was used. In **e**,**g**,**h**,**m**,**p**–**r** a two-sided Student’s *t*-test was used. Source data

**Extended Data Fig. 2:**
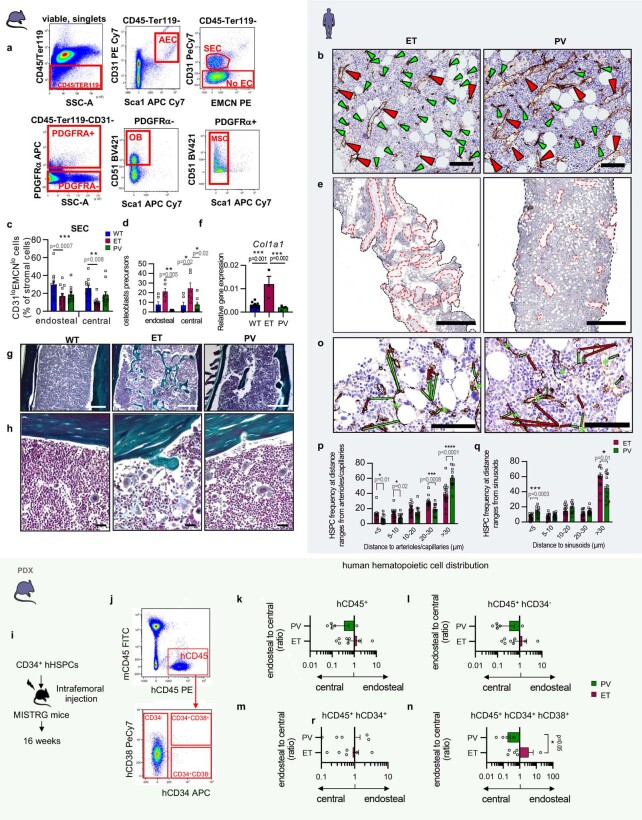
Asymmetric HSC-niche interactions cause differential vascular and stromal remodeling in MPN subtypes. **a**, Gating strategy used for mouse stromal cell populations. CD45^−^Ter119^−^Sca1^+^CD31^+^ arteriolar endothelial cells (AEC), CD45^−^Ter119^−^EMCN^lo^CD31^lo^ sinusoidal endothelial cells (SEC), CD45^−^Ter119^−^CD31^−^ PDGFRα^+^Sca1^−^ mesenchymal stem cells (MSCs), CD45^−^ Ter119^−^CD31^−^PDGFRα^−^Sca1^−^CD51^+^ osteoblast precursors (OBPs). **b**, **e**, **o**, Immunohistochemistry for CD34 and hematoxylin counterstaining in BM trephines from ET or PV patients at baseline. Scale bar, 100μm (**b**, **o**), 1 mm (**e**). **b**, Analysis of the BM vasculature in ET or PV patients. Red arrowheads depict sinusoids and green arrowheads depict arterioles/capillaries. **c**, Frequency of EMCN^lo^CD31^lo^ sinusoidal endothelial cells among CD45^−^Ter119^−^ stromal cells in WT (n = 11 mice), ET- (n = 13 mice) or PV-like (n = 11 mice) animals. **d**, Frequency of PDGFRα^−^Sca1^−^CD51^+^ osteoblast precursors among CD45^−^Ter119^−^CD31^−^ stromal cells in WT (n = 6 mice), ET- (n = 6 mice) or PV-like (n = 7 mice) animals. **e**, Bone area (red dashed line) and tissue area (black dashed line) in ET or PV BM trephine. Scale bar, 1 mm. **f**, *Col1a1* mRNA expression in BM cells from WT (n = 11 mice), ET-like (n = 3 mice) or PV-like (n = 4 mice) animals. **g**-**h**, Trichrome Masson staining of BM sections from WT, ET- or PV-like mice. Scale bar, 200μm. **i**-**n**, Analysis of PDX mice (MISTRG) transplanted with ET (n = 8 mice) or PV (n = 6 mice) hHSPCs, or sham-treated (n = 7 mice). **i**, Outline of the experiment. PV or ET patient-derived CD34+ HSPCs were injected intrafemorally in irradiated MISTRG mice and analyzed 16w post-transplant. **j**, Gating strategy used for human hematopoietic cells. hCD45^+^ pan-human hematopoietic cells, hCD45^+^hCD34^−^ mature hematopoietic cells, hCD45^+^hCD34^+^ hHSPCs, hCD45^+^hCD34^+^hCD38^+^ HPCs, hCD45^+^hCD34^+^hCD38^−^ HSC-enriched cells. **k**-**n**, BM distribution of human MPN cells in the PDX mice. The data represents the ratio of the number of cells in the endosteum over the number of cells in the central marrow. **k**, hCD45^+^ hematopoietic cells. **l**, hCD45^+^hCD34^−^ mature hematopoietic cells. **m**, hCD45^+^hCD34^+^ hHSPCs. **n**, hCD45^+^CD34^+^CD38^+^ HPCs. ET, n = 8 mice; PV, n = 5 mice. **o**, Analysis of the distance between hHSPCs and sinusoids (green lines) and between hHSPCs and arterioles/capillaries (red lines). Green asterisks depict sinusoidal lumen. **p**-**q**, HSPC frequency at distance ranges (μm) from arterioles/capillaries (**p**) or sinusoids (**q**) in MPN human BM trephines. ET, n = 16 patients; PV, n = 14 patients. **c**-**f**, **k**-**n**, Data are means ± SEM. Each square dot is a mouse. **c**-**f**, Two-sided One-way ANOVA. **p**-**q**, **k**-**n**, Two-sided Student’s t-test. *p < 0.05; **p < 0.01; ***p < 0.001. Source data

The dissimilar lodgment and expansion of *JAK2*^V617F^-mutated mHSCs in ET and PV caused differential remodeling of BM vessels. Arterioles and capillaries increased in ET-like mice only (Fig. 2a–d, arrowheads and Extended Data Fig. 2a) and were more abundant in the BM of patients with ET, compared with patients with PV (Fig. 2e and Extended Data Fig. 2b, green arrowheads). In contrast, central BM sinusoids were specifically enlarged in different transgenic^30^ or knockin^33^ models of PV (Fig. 2b, arrows, Fig. 2f,g and Extended Data Fig. 2c) and in human PV, compared with ET (Fig. 2h and Extended Data Fig. 2b, red arrowheads). These results indicate specific remodeling of endosteal and central BM vessels in ET and PV, respectively.

Lodgement of mHSCs near the bone surface was followed by an abnormal expansion of the endosteal BM niche in ET-like mice only. Immunofluorescence staining revealed increased osterix (OSX)^+^ osteoprogenitors and osteoblasts as the probable cause of augmented osteopontin (OPN)^+^ bone formation inside the BM (Fig. 2i–l and Extended Data Fig. 2d). Matching these findings, the bone area was doubled in BM trephines from patients with ET, compared with patients with PV (Fig. 2m and Extended Data Fig. 2e). Increased type I collagen (*Col1a1*) mRNA expression and Masson trichrome staining confirmed the specific expansion of endosteal BM niches in ET (Extended Data Fig. 2f–h). Bone-forming cell expansion was paralleled by increased abundance of bone-resorbing (osteoclastic) cells (Fig. 2n,o), with reportedly reduced osteolytic function due to their *JAK2*^V617F^ mutation^34^, ultimately causing intra-marrow ossification and increased cortical bone volume, bone surface and trabecular separation in ET mice, compared with WT mice (Fig. 2p–r). These results illustrate differential BM stromal remodeling in ET and PV, despite their shared driver mutation (*JAK2*^V617F^).

### hHSPCs reshape their BM niches in MPNs

**Fig. 3:**
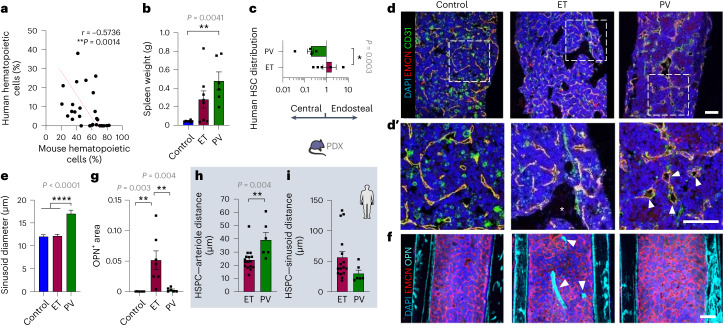
Human HSCs from ET and PV differentially remodel their niche in PDX. **a**–**g**, Analysis of PDX mice (MISTRG recipient mice) transplanted with ET (*n* = 8 mice) or PV (*n* = 6 mice) CD34^+^ hHSPCs intrafemorally, or sham-treated (*n* = 7 mice). Primary cells were isolated from independent ET (*n* = 3) and PV (*n* = 3) donors. **a**, Inverse correlation of the frequencies of human and mouse hematopoietic cells in PDX BM (*n* = 28). The regression linear line is represented by a red dashed line. **b**, Spleen weight in the ET (*n* = 8 mice), PV (*n* = 6 mice) or control (*n* = 7 mice) PDX model. **c**, BM distribution of CD34^+^CD38^−^ hHSCs (ET, *n* = 5 mice; PV, *n* = 4 mice). **d**,**e**, Immunofluorescence of CD31^+^ (green) and EMCN^+^ (red) blood vessels (**d**) and quantification of sinusoid diameter (**e**). Asterisk in **d′** represents bone formation. **f**,**g**, Immunofluorescence of OPN^+^ osteoblasts (turquoise blue) and EMCN^+^ (red) blood vessels (**f**), and quantification of OPN^+^ bone area (**g**); control, *n* = 7 mice; ET, *n* = 7 mice; PV, *n* = 6 mice. Arrowheads in **d′** indicate sinusoids; arrowheads in **f** indicate OPN^+^ area (bone formation). Scale bar in **d**, **d′** and **f**, 200 µm. **h**,**i**, Analysis of ET (*n* = 16 patients) or PV (*n* = 6 patients) human BM trephines (blue background). Mean distance between CD34^+^ hHSPC and the closest arteriole or capillary (**h**) or sinusoid (**i**). Each dot represents one individual. Each square dot represents a mouse or individual. Data are shown as the mean ± s.e.m. *P < 0.05; **P < 0.01; ***P < 0.001; ****P < 0.0001. In **a** a two-sided Spearman correlation test was used. In **b**,**e**,**g** a two-sided, one-way ANOVA was used. In **c**,**h**,**i** a two-sided Student’s *t*-test was used. Source data

To validate these observations in hHSCs, a patient-derived xenograft (PDX) model of PV and ET was established by transplanting hCD34^+^ HSPCs from patients with ET or PV intrafemorally into sublethally irradiated MISTRG mice^35^ (Extended Data Fig. 2i). Sixteen weeks after transplantation, the abundance of human hematopoietic cells was negatively correlated with the frequency of mouse hematopoietic cells (Fig. 3a), suggesting interspecies HSC competition for niche occupancy. The expansion of human MPN cells caused splenomegaly, which was more pronounced in mice transplanted with PV hHSPCs (Fig. 3b), reproducing the clinical observation. In mice with engraftment >1% hCD45^+^ cells, hHSCs could be reliably measured and were asymmetrically distributed in PDX models (Fig. 3c). Mimicking MPN mice, the differential location affected CD34^+^ hHSPCs, but not the more committed CD34^−^ cells (Extended Data Fig. 2j–n). Resembling MPN mice, sinusoidal vasodilation was observed in mice engrafted with PV hHSPCs (Fig. 3d,e), whereas the bone (OPN^+^) area was 25-fold higher in mice engrafted with ET hHSPCs, compared with PV hHSPCs (Fig. 3f,g). The differential vascular and stromal remodeling probably followed hHSPC expansion in each niche: in ET, hHSPCs were found in contact to arterioles and capillaries, which are abundant near bone in humans^36^ and support developmental bone growth in mice^37^, whereas a perisinusoidal hHSPC location appeared more frequently in human PV (Fig. 3h,i and Extended Data Fig. 2o–q). Therefore, dissimilar interactions of mutant hHSCs carrying the same oncogenic driver (*JAK2*^V617F^) with their BM niches cause differential tissue remodeling.

### Similar niche remodeling in ET regardless of driver mutation

**Extended Data Fig. 3:**
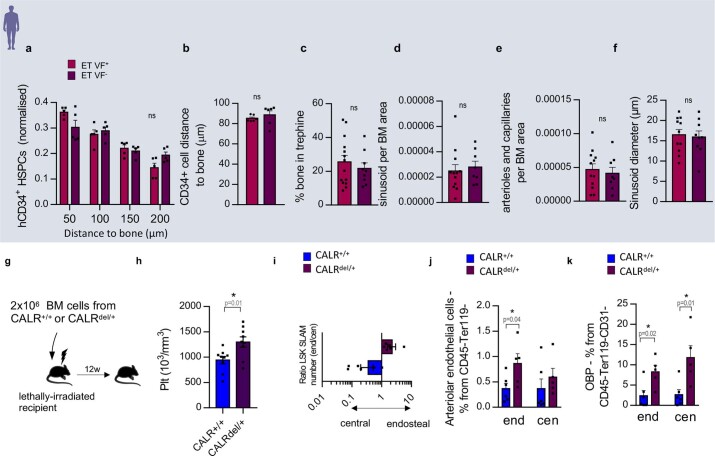
*JAK*2^V617F+^ and *JAK*2^V617F−^ ET patients and mice display similar HSPC distribution and microenvironmental alterations. **a**, **b**, Distribution of the minimal distance (**a**) or mean distance (**b**) between CD34^+^ hHSPCs and the bone surface following the analysis of immunohistochemistry for CD34 and hematoxylin counterstaining of BM trephines from *JAK2*^*V617F*^ positive (VF^+^) or negative (VF^−^) human essential thrombocythemia (ET; n = 5-6 patients). **c**, Percentage of BM trephine occupied by bone in VF^+^ (n = 14 patients) or VF^−^ (n = 9 patients) human ET. **d**, Sinusoids per BM area in VF^+^ (n = 12 patients) or VF^−^ (n = 8 patients) human ET. **e**, Number of arterioles/capillaries per BM area in VF^+^ (n = 12 patients) or VF^−^ (n = 8 patients) human ET. **f**, Mean sinusoid diameter in VF^+^ (n = 11) or VF^−^ (n = 9) human ET. **g**, Outline of CALR-mutant ET model. 2×10^6^ BM cells from of ET-like (*CALR*^*del/+*^) or WT control (*CALR*^*+/+*^) mice were i.v. injected into lethally-irradiated WT recipients (n = 6 mice) that were analyzed 12w later (**h**-**k**). **h**, Circulating platelets in ET-like (*CALR*^*del/+*^, n = 9) or WT control (*CALR*^*+/+*^, n = 9 mice) chimeric mice. **i**, BM distribution (endosteal:central ratio) of Lin^−^ Sca1^+^cKit^+^ (LSK) CD48^−^CD150^+^ HSCs (n = 6 mice). **j**, Frequency of Sca1^hi^CD31^hi^ arteriolar endothelial cells among CD45^−^Ter119^−^ stromal cells. WT, n = 6 mice; ET, n = 5 mice. **k**, Frequency of CD51^+^PDGFRα^−^ osteoblast progenitors (OBP) among CD45^−^Ter119^−^CD31^−^ stromal cells. WT, n = 6 mice; ET, n = 5 mice. **a-k**, Data are means ± SEM. Each square dot is a mouse. Two-sided Student’s t-test, *p < 0.05. Source data

We tested whether the HSPC distribution and microenvironmental changes observed in patients with ET and ET-like mice were restricted to those carrying the *JAK2*^V617F^ mutation. HSPC distance to the bone surface, bone area and vessel numbers were indistinguishable in *JAK2*^V617F+^ and *JAK*2^V617F^^−^ patients with ET (Extended Data Fig. 3a–f). For confirmation, we examined a *JAK*2^V617F^^−^ ET-like mouse model carrying the most common *CALR* mutation^38^. *CALR*^del/+^ mice with phenotypic disease recapitulated key features of *JAK*2^V617F+^ ET-like mice, including asymmetric HSC distribution and expansion of endosteal arteriolar endothelial cells and osteoblast precursors (Extended Data Fig. 3g–k). Therefore, HSC distribution and niche remodeling are similarly affected in ET, regardless of the *JAK*2^V617F^ mutation.

### Enforced ET HSC location in the central niche aggravates MPN

**Fig. 4:**
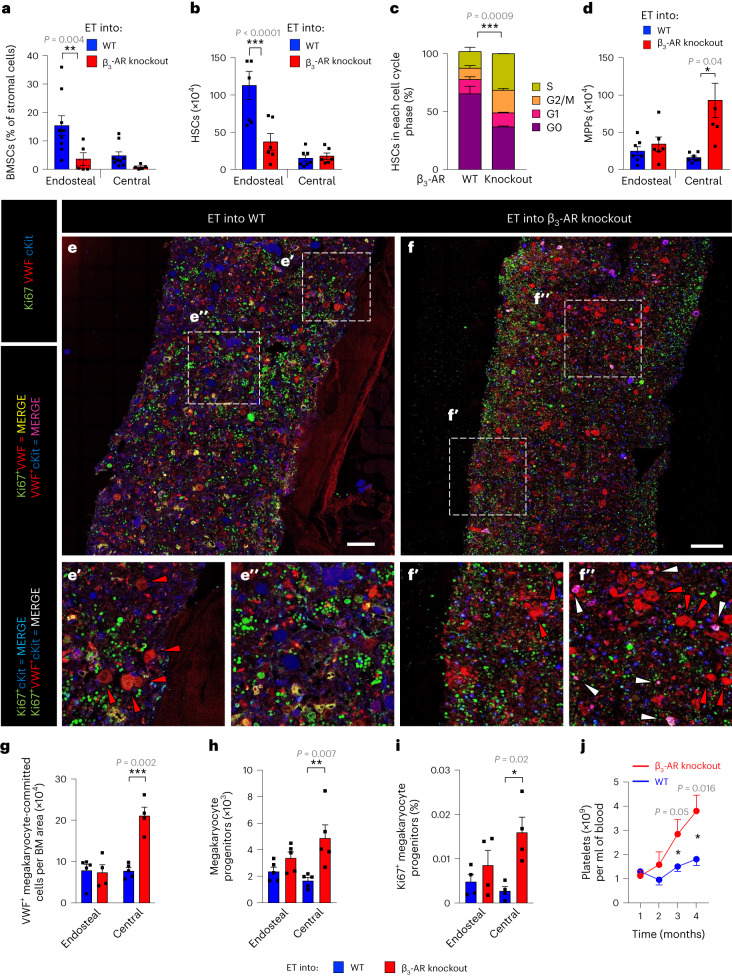
ET relocation to the central niche worsens disease development. **a**–**d**, Analysis of the endosteal and central BM of β_3_-AR knockout mice (*n* = 5 mice) or WT (*n* = 7 animals) mice 8 weeks after transplantation with BM cells from *iVav-Cre;Jak2*^V617F^ ET-like mice. **a**, Frequency of PDGFRα^+^Sca1^−^ BMSCs among CD45^−^Ter119^−^CD31^−^ stromal cells. WT, *n* = 7 mice; β_3_-AR knockout, *n* = 5 mice. **b**, LSK CD48^−^CD150^+^ HSCs in the endosteal or central BM. WT, *n* = 7 mice; β_3_-AR knockout, *n* = 5 mice. **c**, Cell cycle analysis showing reduced frequency of quiescent (G0) HSCs in the central BM of β_3_-AR knockout (*n* = 6) or WT (*n* = 5) mice. The gating strategy is shown in Extended Data Fig. 1a. **d**, LSK CD48^+^CD150^−^ MPPs in the endosteal or central BM. WT, *n* = 5 mice; β_3_-AR knockout, *n* = 6 mice. **e**–**j**, Analysis of the endosteal and central BM of β_3_-AR knockout mice (*n* = 6 mice, unless indicated otherwise) or WT mice 16 weeks after transplantation with BM cells from *iVav-Cre;Jak2*^V617F^;*VWF-TdTomato* ET-like mice, to detect megakaryocyte-committed cells through VWF expression. **e**,**f**, Immunofluorescence of Ki67 (green), VWF-tdTomato (VWF, red), cKit (blue). **e′**, **e″** and **f′**, **f″** represent high magnification insets of the endosteal (**e′**, **f′**) and central (**e″**, **f″**) BM. The red arrowhead indicates megakaryocytes; the white arrowhead indicates proliferative megakaryocyte progenitors. Scale bar, 100 µm. **g**, Number of VWF^+^ megakaryocyte-committed cells. WT, *n* = 5 mice; β_3_-AR knockout, *n* = 4 mice. **h**, Number of LSK^+^CD150^+^CD41^+^ granulocyte-macrophage, erythrocyte and megakaryocyte progenitors. WT, *n* = 5 mice; β_3_-AR knockout, *n* = 5 mice. **i**, Frequency of VWF^+^cKit^+^Ki67^+^ proliferative megakaryocyte progenitors. WT, *n* = 4 mice; β_3_-AR knockout, *n* = 4 mice. **j**, Circulating platelets measured by blood counter 1–4 months after transplantation. WT, *n* = 6 mice; β_3_-AR knockout, *n* = 6 mice. In **a**–**d**,**g**–**i** each square dot is a mouse. The gating strategy is shown in Extended Data Figs. 1 and 2. Data are shown as the mean ± s.e.m. *P < 0.05; **P < 0.01; ***P < 0.001. In **a**,**b**,**d**,**g**–**i** a two-sided one-way ANOVA was used. In **c**,**j** a two-sided Student’s *t*-test was used. Source data

**Extended Data Fig. 4:**
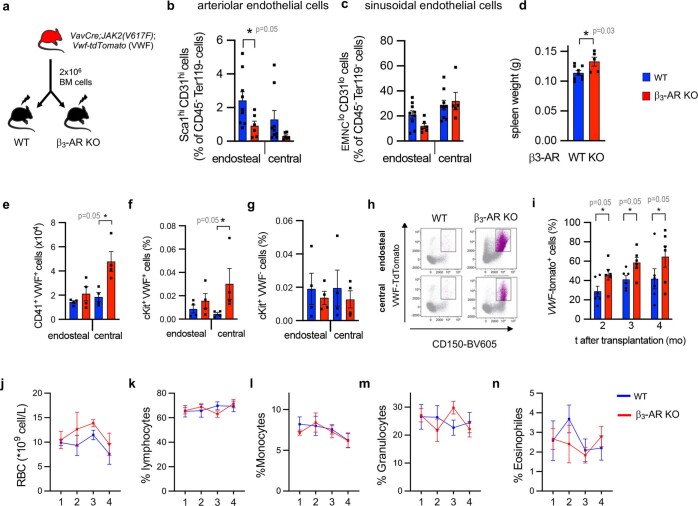
Prematurely aged β_3_-AR knock-out niche worsens MPN development. **a**-**i**, Analysis of WT mice (n = 9 mice) or β_3_-AR knock-out mice (n = 6 mice) 8w after transplantation of BM cells from *iVav-Cre;JAK2*^*V617F*^ ET-like mice. **a**, Outline of the experiment. **b**, Frequency of Sca1^hi^CD31^hi^ arteriolar endothelial cells among CD45^−^Ter119^−^ stromal BM cells. **c**, Frequency of EMCN^lo^CD31^lo^ sinusoidal endothelial cells among CD45^−^Ter119^−^ stromal BM cells. **d**, Spleen weight of WT (n = 9 mice) or β_3_-AR KO (n = 5 mice) animals. **e**-**h**, Analysis of the endosteal and central BM of β_3_-AR knock-out mice or WT mice 16w after transplantation with BM cells from *iVav-Cre;JAK2*^*V617F*^;*Vwf-TdTomato* ET-like mice, to detect megakaryocyte-committed cells through Von Willebrand factor (VWF) expression. **e**, BM CD41^+^VWF^+^ megakaryocyte-committed cells (n = 5 mice). **f**-**g**, BM frequency of (**f**) megakaryocyte-committed cKit^+^VWF^+^ or (**g**) -non-committed cKit^+^VWF^−^ HSPCs (n = 4 mice). **h**, Flow cytometry diagram showing CD150^+^VWF^+^ megakaryocyte progenitors among BM Lin^−^Sca1^−^cKit^+^ cells. **i**, Frequency of circulating VWF^+^ megakaryocyte committed cells 2-4 months after transplantation (n = 6 mice). **j**-**n**, Blood counts 1–4 months after transplantation (n = 6 mice). **j**, Red blood cells. **k**, Lymphocytes. **l**, Monocytes. **m**, Granulocytes. **n**, Eosinophiles. **b**-**g**, **i**-**n**, Data are means ± SEM. **b**-**i**, Each square dot is a mouse. Two-sided Student’s t-test, *p < 0.05. Source data

The endosteal niche is rich in arterioles and capillaries, and promotes HSC quiescence^17,20,22,39–42^, while active HSCs transmigrate through sinusoids, which are abundant in central BM^24^. Therefore, we hypothesized that the comparatively more restrictive endosteal niche might contribute to explain why ET is generally less aggressive than PV, which preferentially expands in the more permissive central BM niche. Thus, we tested whether experimental interference with the HSC-niche interaction might impact ET progression. Reduced activation of the β_3_-adrenergic receptor (AR) by sympathetic nerve fibers worsens PV progression^43^ and promotes myeloid cell expansion during aging^28^. Lack of β_3_-AR reduces endosteal BM niches and their HSCs, while expanding central BM niches already at adulthood^28^. Therefore, we used β_3_-AR-deficient mice to enforce the interaction of ET cells, which normally expand in endosteal niches, with central BM niches. To genetically trace megakaryocyte lineage cells, which preferentially expand in ET, we intercrossed ET-like mice with transgenic mice expressing a tdTomato fluorescent reporter under the regulatory elements of Von Willebrand factor (VWF)^44^, and transplanted their BM cells into β_3_-AR-deficient or WT mice (Extended Data Fig. 4a). In β_3_-AR knockout mice, endosteal vessels were halved, reproducing in MPN the premature microenvironmental aging features of β_3_-AR-deficient mice^28^ (Extended Data Fig. 4b,c). In β_3_-AR knockout mice, endosteal BM mesenchymal stem cells (BMSCs) and HSCs were reduced two- to threefold (Fig. 4a,b), while HSCs proliferated more and multipotent progenitors (MPPs) expanded in the central BM, associated with increased spleen infiltration (Fig. 4c,d and Extended Data Fig. 4d). Multicolor immunofluorescence combined with high-throughput analysis 5 months after transplantation showed a 2.5-fold expansion of megakaryocyte-committed (VWF-tdTomato^+^) cells specifically in the central (not endosteal) BM of β_3_-AR knockout mice (Fig. 4e–g). This result was confirmed by flow cytometry using CD41 as an independent marker of megakaryocyte commitment (Extended Data Fig. 4e). Expansion of megakaryocytic lineage cells in the central niche was explained by increased central BM frequency of myeloid and megakaryocyte progenitors (VWF-tdTomato^+^; Extended Data Fig. 4f–h). This was probably the consequence of granulocyte-monocyte, erythroid, megakaryocyte progenitor expansion in the central BM of β_3_-AR-deficient mice due to their sixfold higher proliferation rate (Fig. 4h,i). Consequently, disease acceleration was observed in β_3_-AR knockout mice, with circulating megakaryocyte-committed cells and platelets rising faster (Fig. 4j and Extended Data Fig. 4i). Erythrocytosis was not observed (Extended Data Fig. 4j–n), implying that the relocation to the central BM niche increases ET aggressiveness, but does not switch it to PV. Therefore, MPN progression is worsened in a prematurely aged BM microenvironment, suggesting that the specialized niche hosting mutant cells can modulate their expansion.

### Ruxolitinib restores the endosteal niche and HSC quiescence in PV

**Fig. 5:**
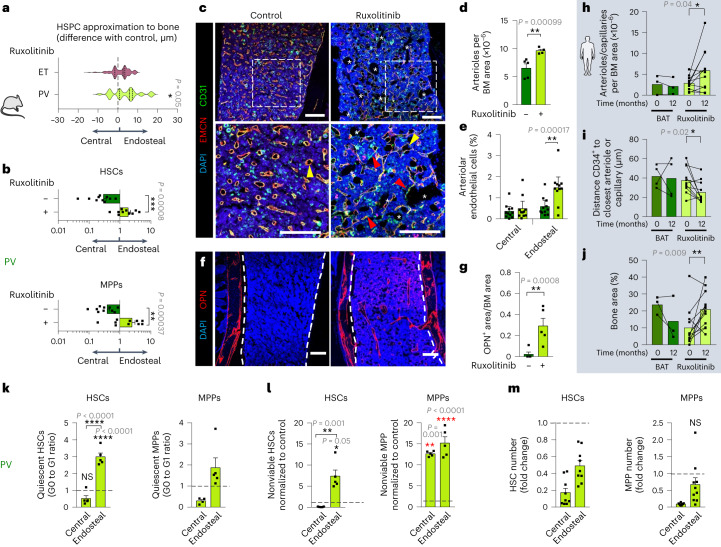
JAK inhibition restores the endosteal niche and HSC quiescence in PV. **a**, Distance between HSPCs and the bone surface measured through intravital microscopy (Extended Data Fig. 1m) 3 days after ruxolinitib treatment (70 mg kg^−1^, once daily, normalized to vehicle-treated control mice). PV, *n* = 144 cells; ET, *n* = 146 cells. HSPCs were pooled from three independent experiments. **b**–**g**,**k**–**m**, WT mice were lethally irradiated, transplanted with BM cells from PV-like mice and treated with ruxolitinib (70 mg kg^−1^, once daily, three times weekly) or vehicle for 5 weeks (outline shown in Extended Data Fig. 5a). **b**, BM distribution of LSK CD48^−^CD150^+^ HSCs (top) or LSK CD48^+^CD150^−^ MPPs (bottom) in PV-like mice. Data are the ratio between endosteal and central BM cells; control, *n* = 10 mice; ruxolitinib, *n* = 10 mice. The gating strategy is shown in Extended Data Fig. 1a. **c**,**d**, Immunofluorescence of CD31^+^ (green) and EMCN^+^ (red) blood vessels. **c**, Representative images. Nuclei were counterstained with DAPI (blue). Asterisks represent area occupied by bone; yellow arrowheads indicate sinusoids; and red arrowheads depict arterioles. Scale bar, 100 µm. **d**, Quantification of arterioles per mouse BM area; control, *n* = 5 mice; ruxolitinib, *n* = 4 mice. **e**, Frequency of Sca1^hi^CD31^hi^ arteriolar endothelial cells among CD45^−^Ter119^−^ stromal cells; control, *n* = 11 mice; ruxolitinib, *n* = 11 mice. **f**,**g**, Immunofluorescence (**f**) and quantification (**g**) of OPN^+^ osteoblasts (red). Nuclei were counterstained with DAPI (blue); control, *n* = 5 mice; ruxolitinib, *n* = 5 mice. Scale bar, 100 µm. Dashed line depicts the interface between bone and BM. **h**–**j**, Longitudinal analysis of paired BM trephines from patients with PV before or 12 months after treatment with ruxolitinib or BAT. **h**, Arterioles or capillaries per human BM area; BAT, *n* = 3 samples; ruxolitinib, *n* = 11 samples. **i**, Shortest distance between CD34^+^ hHSPCs and arterioles or capillaries. BAT, *n* = 4 samples; ruxolitinib, *n* = 11 samples. **j**, BM area occupied by bone; BAT, *n* = 3 samples; ruxolitinib, *n* = 12 samples. **k**, Ratio (G0 to G1) of quiescent HSCs (left) and MPPs (right) in the endosteal or central BM; control, *n* = 5 mice; ruxolitinib, *n* = 5 mice. NS, not significant. **l**, Frequency of nonviable (sub-G0) HSCs (left) and MPPs (right); control, *n* = 5 mice; ruxolitinib, *n* = 5 mice. **m**, Fold change of HSCs (left) and MPPs (right) after ruxolitinib treatment; control, *n* = 9 mice; ruxolitinib, *n* = 9 mice. The black horizontal dashed line in **k**, **l** and **m** marks the normalized control. In **a**,**b**,**d**,**e**,**g**–**m** each square dot is a mouse or individual. Data are shown as the mean ± s.e.m. *P < 0.05; **P < 0.01; ***P < 0.001; ****P < 0.0001. In **a**,**b**,**d**,**g**,**k**–**m** a two-sided Student’s *t*-test was used. In **h**–**j** a paired two-sided Student’s *t*-test was used. In **e** a two-sided, one-way ANOVA was used. Source data

**Extended Data Fig. 5:**
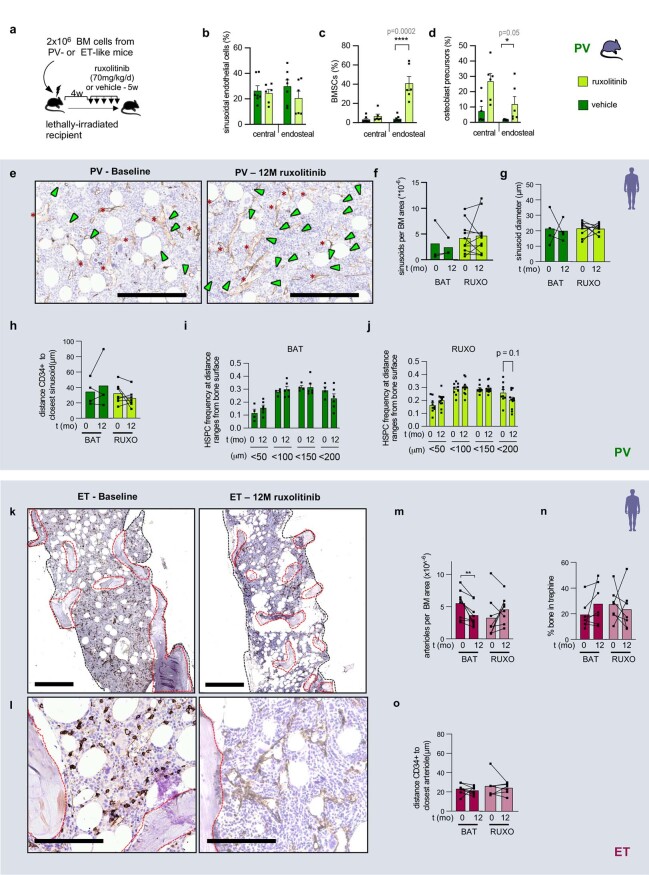
Different effects of JAK inhibitor on HSPC-niche interactions in MPN subtypes. **a**-**d**, WT mice were lethally irradiated, transplanted with BM cells from PV-like or ET-like mice and treated 4w post-transplantation with ruxolitinib (70 mg/kg, o.d.,3/w) or vehicle for 5w. (control, n = 7 mice; ruxolitinib, n = 6 mice). **a**, Outline of the experiment. **b**, Frequency of EMCN^lo^CD31^lo^ sinusoidal endothelial cells among CD45^−^Ter119^−^ BM cells. **c**, Frequency of PDGFRα^+^Sca1^−^ BM mesenchymal stem cells (BMSCs) among CD45^−^Ter119^−^CD31^−^ stromal cells. **d**, Frequency of PDGFRα^−^Sca1^−^CD51^+^ osteoblasts precursors among CD45^−^Ter119^−^CD31^−^ stromal cells in PV-like mice. **e**-**j**, Analysis of PV patients’ paired BM trephines at baseline or after 12-month treatment with ruxolitinib or best available therapy (BAT). **e**, Immunohistochemistry for CD34 and hematoxylin counterstaining in BM trephines from the same PV patient at baseline or 12mo after ruxolitinib treatment. Red arrowheads depict sinusoids and green arrowheads depict arterioles/capillaries. Scale bar, 100μm. **f**, Sinusoids per BM area (BAT, n = 3 patients; ruxolitinib, n = 11 patients). **g**, Mean sinusoid diameter (BAT, n = 4 patients; ruxolitinib, n = 9 patients). **h**, Mean distance between CD34^+^ HSPCs and the closest sinusoid (BAT, n = 4 patients; ruxolitinib, n = 7 patients). **i**-**j**, Distribution of CD34^+^ HSPC frequency of distance ranges (μm) from the bone surface in paired samples from the same PV patients at baseline or after 12- month treatment with BAT (**i**, n = 4 patients) or ruxolitinib (**j**, n = 12 patients). **k**-**o**, Immunohistochemistry for CD34 and hematoxylin counterstaining of paired samples from the same ET patients at baseline or after 12-month treatment with ruxolitinib or BAT. **k**-**l**, Bone area (red dash line) and tissue area (black dash line) in ET paired BM trephine. Scale bar, 1 mm (**k**), 100μm (**l**). **m**, Arterioles/capillaries per BM area (BAT, n = 9 patients; ruxolitinib, n = 8 patients). **n**, Bone area (BAT, n = 7 patients; ruxolitinib, n = 7 patients). **o**, Distance between CD34^+^ HSPCs and the closest arteriole/capillary (BAT, n = 8 patients; ruxolitinib, n = 7 patients). **b**-**d**, **f**-**j**, **m**-**o**, Data are means ± SEM. Each square dot is a mouse or individual. Two-sided Student’s t-test, *p < 0.05; **p < 0.01; ****p < 0.0001. Source data

To gain insight into how oncogenic JAK–STAT signaling affects HSPC-niche interactions, we treated MPN mice with the JAK1/2 inhibitor ruxolitinib or control vehicle. Ruxolitinib treatment of mice transplanted with PV cells triggered the endosteal lodgment of HSCs and MPPs (Fig. 5a,b and Extended Data Fig. 5a). Consequently, endosteal arterioles increased, while central sinusoids were unchanged (Fig. 5c–e and Extended Data Fig. 5b). Endosteal vessel expansion was accompanied by BMSC and osteoblast precursor buildup in PV-like mice, increasing the BM area occupied by bone (Fig. 5f,g and Extended Data Fig. 5c,d). Consistent results were observed in humans: one-year treatment with ruxolitinib (but not with best available therapy (BAT)) doubled BM arterioles and capillaries, and bone area, and hHSPCs became closer to arterioles and capillaries (not sinusoids; Fig. 5h–j and Extended Data Fig. 5e–j).

Endosteal HSC relocation and niche expansion after ruxolitinib treatment was associated with sixfold-increased quiescence of endosteal (not central) HSCs in PV mice (Fig. 5k). Additionally, cell death increased by 5–14-fold, leading to more than tenfold-reduced PV-like HSCs and MPPs (Fig. 5l,m). These results are consistent with, and help to explain, the therapeutic effects of ruxolitinib in patients with PV^14,15^.

### Ruxolitinib expands central BM MPPs in ET

**Fig. 6:**
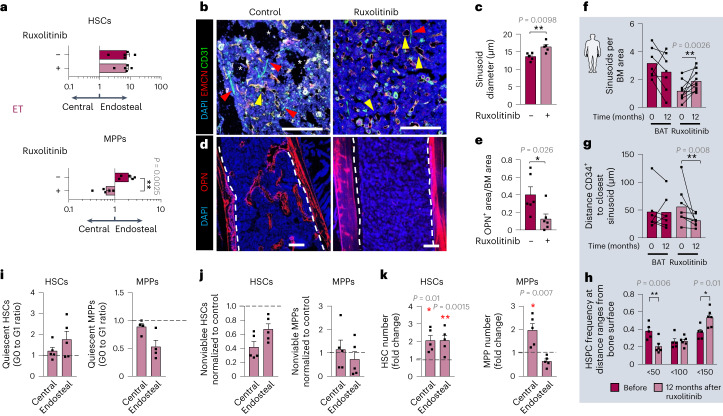
JAK inhibitor expands central BM MPPs in ET. **a**–**e**,**i**–**k**, WT mice were lethally irradiated, transplanted with BM cells from ET-like mice and treated with ruxolitinib (70 mg kg^−1^, once daily, three times weekly) or vehicle for 5 weeks (outline shown in Extended Data Fig. 5a). **a**, BM distribution of LSK CD48^−^CD150^+^ HSCs (top) or LSK CD48^+^CD150^−^ MPPs (bottom). Data are the ratio between endosteal and central BM cells; control, *n* = 5 mice; ruxolitinib, *n* = 5 mice. The gating strategy is shown in Extended Data Fig. 1a. **b**,**c**, Immunofluorescence of CD31^+^ (green) and EMCN^+^ (red) blood vessels. **b**, Representative images. Nuclei were counterstained with DAPI (blue). Asterisks represent area occupied by bone; yellow arrowheads indicate sinusoids; and red arrowheads depict arterioles. Scale bar, 100 µm. **c**, Quantification of sinusoid diameter; control: *n* = 5 mice; ruxolitinib, *n* = 4 mice. **d**,**e**, Immunofluorescence (**d**) and quantification (**e**) of OPN^+^ osteoblasts (red). Nuclei were counterstained with DAPI (blue); control, *n* = 5 mice; ruxolitinib, *n* = 5 mice. Scale bar, 100 µm. Dashed line depicts the interface between bone and BM. **f**–**h**, Longitudinal analysis of paired BM trephines from patients with ET before or 12 months after treatment with ruxolitinib or BAT. **f**, Sinusoids per BM area; BAT, *n* = 7 samples; ruxolitinib, *n* = 10 samples. **g**, Shortest distance between CD34^+^ hHSPCs and sinusoids; BAT, *n* = 7 samples; ruxolitinib, *n* = 8 samples. **h**, Frequencies of CD34^+^ hHSPCs at distance ranges from the bone surface before (*n* = 5 samples) or 12 months after ruxolitinib treatment (*n* = 6 samples). **i**, Ratio (G0 to G1) of quiescent HSCs (left, *n* = 5 mice) and MPPs (right, *n* = 4 mice) in the endosteal or central BM. **j**, Frequency of nonviable (sub-G0) HSCs (left, *n* = 5 mice) and MPPs (right, *n* = 5 mice). **k**, Fold change of HSCs (left, *n* = 5 mice) and MPPs (right, *n* = 5 mice) after ruxolitinib treatment. The black horizontal dashed line in **i**, **j** and **k** marks the normalized control. Each square dot is a mouse or individual. Data are shown as the mean ± s.e.m. *P < 0.05; **P < 0.01. In **a**,**c**,**e**,**f**–**h**,**i**–**k** a two-sided Student’s *t*-test was used. Source data

Contrastingly, chronic ruxolitinib treatment in ET-like mice did not affect the (already) preferential endosteal HSC location but, unexpectedly, it relocated MPPs to the central BM (Fig. 6a). Consequently, central sinusoids were enlarged (Fig. 6b,c), whereas the endosteal niche was reduced (Fig. 6d,e). These results were validated in humans, as sinusoids (not arterioles or capillaries) increased, and HSPCs were displaced from the endosteum and located closer to sinusoids in ruxolitinib-treated patients with ET (Fig. 6f–h and Extended Data Fig. 5k–o). Contrasting findings were observed in PV (Fig. 5k–m): in ET-like mice ruxolitinib did not affect HSC quiescence or survival, and was associated with doubled HSCs and MPPs (Fig. 6i–k). These results suggest that dissimilar HSC-niche interactions underlie a distinctive response to JAK inhibitor, possibly explaining the more pronounced therapeutic effects of ruxolitinib in patients with PV (compared with patients with ET)^13–15^.

**Extended Data Fig. 6:**
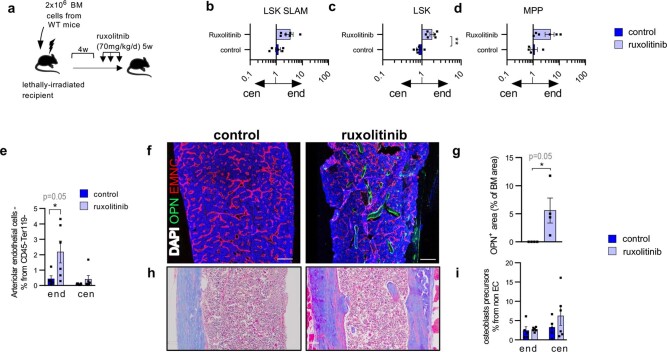
Effects of JAK inhibitor on HSPC-niche interactions in WT mice. **a**-**i**, WT mice were lethally irradiated, transplanted with BM cells from WT mice (for comparison with mice transplanted with MPN cells) and treated 4w post transplantation with ruxolitinib (70 mg/kg, o.d., 3/w) or vehicle for 5w. **a**, Outline of the experiment. **b**-**d**, BM distribution of the different cell populations in WT mice following chronic treatment with ruxolitinib (n = 6 mice) or vehicle (n = 5 mice). The data represents the ratio of endosteal and central BM cells. **b**, Lin^−^Sca1^+^cKit^+^ (LSK) CD48^−^CD150^+^ HSCs. **c**, LSK HSPCs. **d**, LSK CD48^+^CD150^−^ multipotent progenitors (MPPs). **e**, Frequency of Sca1^hi^CD31^hi^ arteriolar endothelial cells among CD45^−^Ter119^−^ BM stromal cells in WT mice treated with ruxolitinib (n = 6 mice) or vehicle (n = 5 mice). **f**, Immunofluorescence of osteopontin (OPN)^+^ osteoblasts (green) and endomucin (EMCN)^+^ blood vessels (red). Scale bar, 200μm. **g**, Quantification of OPN^+^ bone area (n = 4 mice). **h**, Trichrome Masson staining of BM sections. **i**, Frequency of PDGFRα^−^Sca1^−^CD51^+^ osteoblast precursors among CD45^−^Ter119^−^CD31^−^ stromal cells in WT mice treated with ruxolitinib (n = 6 mice) or vehicle (n = 5 mice). **b-e, g**-**i**, Data are means ± SEM. Each square dot is a mouse. Student’s t-test; *p < 0.05, **p < 0.01. Source data

Because ruxolitinib can inhibit both mutant and non-mutated *JAK2*, we investigated its impact on WT mice (Extended Data Fig. 6a). Five-week ruxolitinib treatment fostered the endosteal lodgment of HSCs and MPPs (Extended Data Fig. 6b–d), leading to increased endosteal arteriolar endothelial cells, osteoblast precursors and bone area (Extended Data Fig. 6e–i). Thus, ruxolitinib may affect the distribution of unmutated HSCs and their normal microenvironment.

### CDC42 regulates niche location and proliferation of MPN HSCs

**Fig. 7:**
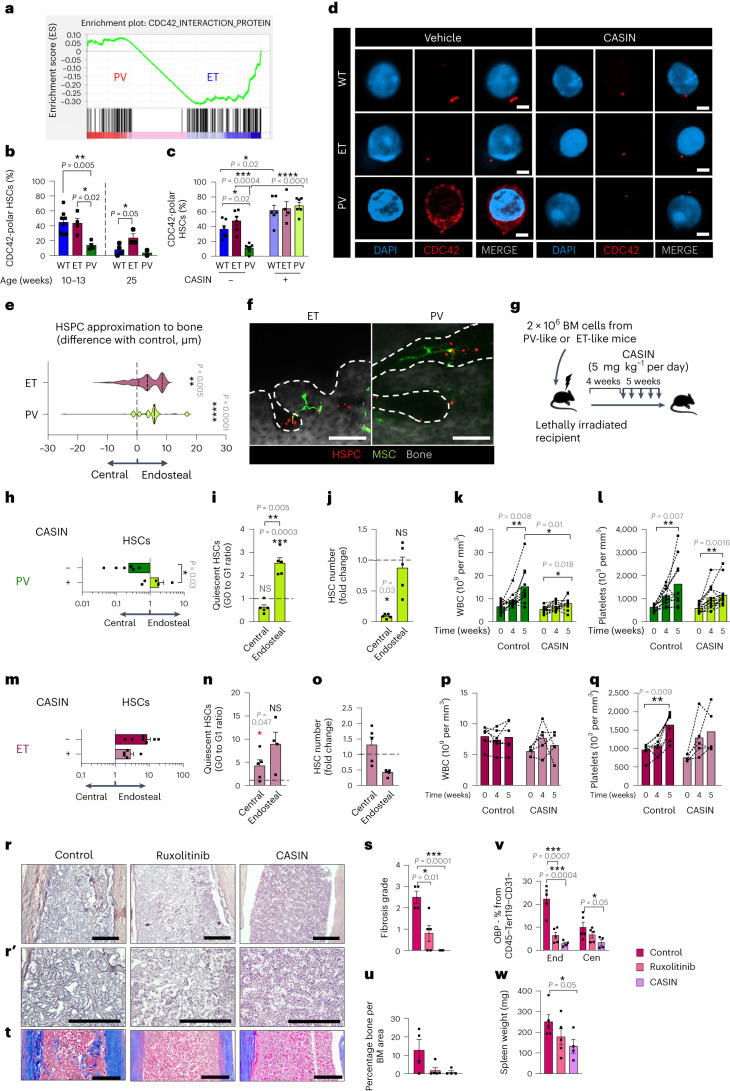
CDC42 polarity regulates the location and proliferation of MPN HSCs. **a**, Gene set enrichment analysis of 187 CDC42-interacting proteins (Extended Data Fig. 7) in an hHSC RNA-seq dataset^45^, including PV and ET. **b**–**d**, CDC42 immunofluorescence (red) and frequencies of CDC42-polar LSK CD48^−^CD150^+^ HSCs from WT or MPN mice. **b**, Fluorescence-activated cell-sorted HSCs from 10–13 or 25-week-old WT, ET-like or PV-like mice were cultured for 16 h on fibronectin-coated imaging slides, stained for CDC42 and imaged. 10–13-week-old mice: WT, *n* = 8; ET, *n* = 4; PV, *n* = 4; 25-week-old mice: WT, *n* = 4; ET, *n* = 3; PV, *n* = 2. *n* is the number of independent experiments. **c**, Fluorescence-activated cell-sorted HSCs from 10–13-week-old WT, ET-like and PV-like mice were treated in vitro for 16 h with the CDC42 inhibitor CASIN (1 µm) or vehicle, stained for CDC42 and imaged. Without CASIN: WT, *n* = 6; ET, *n* = 6; PV, *n* = 7. With CASIN: WT, *n* = 6; ET, *n* = 4; PV, *n* = 6. *n* is the number of independent experiments. **d**, Representative images of the HSCs in Fig. 7c. **e**,**f**, HSCs were sorted from DsRed ET-like or PV-like donor mice and injected intravenously into lethally irradiated *Nes-GFP* recipients subsequently treated with CASIN (10 mg kg^−1^ per day) and analyzed by intravital imaging after 3 days (*n* = 3 independent experiments). **e**, Distance between HSPCs and the bone surface in CASIN-treated recipients, normalized to vehicle-treated control from three different experiments. PV, *n* = 153 cells; ET, *n* = 147 cells. **f**, Z-stacks of *Nes-GFP*^+^ skull BM 3 days after transplantation and CASIN treatment. HSPCs (red), GFP^+^ (green) niche cells and the bone signal (dashed line) from secondary-harmonic generation from collagen (gray) are shown. Scale bar, 100 µm. **g**, Outline of the experiment. BM cells from ET-like or PV-like mouse were transplanted in irradiated WT recipients subsequently treated for 5 weeks with CASIN (5 mg kg^−1^ per day) or vehicle, starting 4 weeks after transplant. **h**–**q**, Transplant of PV-like (**h**–**l**) or ET-like (**m**–**q**) BM cells in WT recipient mice treated chronically with the CDC42 inhibitor CASIN. **h**–**j**, Control, *n* = 5 and 6; CASIN, *n* = 5. **k**,**l**, Control, *n* = 11; CASIN, *n* = 13. **m**–**q**, Control, *n* = 4–7; CASIN, *n* = 5. *n* is the number of independent experiments. **h**,**m**, BM HSC distribution expressed as the ratio of endosteal to central HSCs in PV-like (**h**) or ET-like (**m**) mice treated with CASIN or vehicle. **i**,**n**, Frequency of quiescent (G0 to G1 ratio) HSCs in the endosteal or central BM of PV-like (**i**) or ET-like (**n**) mice. **j**,**o**, Fold change of HSCs in the endosteal or central BM of PV-like (**j**) or ET-like (**o**) mice after CASIN treatment. The black horizontal dashed line in **i**, **j**, **n** and **o** marks the normalized control. **k**,**p**, WBCs before and 4 or 5 weeks after CASIN treatment of PV-like (**k**) or ET-like (**p**) mice. **l**,**q**, Circulating platelets before and 4 or 5 weeks after CASIN treatment of PV-like (**l**) or ET-like (**q**) mice. **r**–**w**, WT mice were lethally irradiated, transplanted with BM cells from ET-like mice and treated 12 weeks after transplantation (at secondary myelofibrosis stage) with ruxolitinib (70 mg kg^−1^, once daily, three times weekly), CASIN (5 mg kg^−1^ per day) or vehicle for 5 weeks. **r**,**s**, Reticulin fibers staining (**r**) and fibrosis grade (**s**). Control, *n* = 4 mice; ruxolitinib, *n* = 5 mice; CASIN, *n* = 4 mice. Scale bar, 200 µm. **t**,**u**, Trichrome Masson staining (**t**) and osteosclerosis quantification (**u**). Control, *n* = 4 mice; ruxolitinib, *n* = 5 mice; CASIN, *n* = 4 mice. Scale bar, 200 µm. **v**, Frequency of PDGFRα^−^Sca1^−^CD51^+^osteoblast precursors among CD45^−^Ter119^−^CD31^−^ stromal cells in myelofibrotic mice treated with ruxolitinib (*n* = 5 mice), CASIN (*n* = 4 mice) or vehicle (*n* = 5 mice). **w**, Spleen weight in myelofibrotic mice treated with ruxolitinib (*n* = 5 mice), CASIN (*n* = 4 mice) or vehicle (*n* = 5 mice). In **b**,**c**,**e**,**h**–**q**,**s**–**w** data are shown as the mean ± s.e.m. Each square dot is a mouse. *P < 0.05; **P < 0.01; ***P < 0.001; ****P < 0.0001. A two-sided Student’s *t*-test was used. Source data

**Extended Data Fig. 7:**
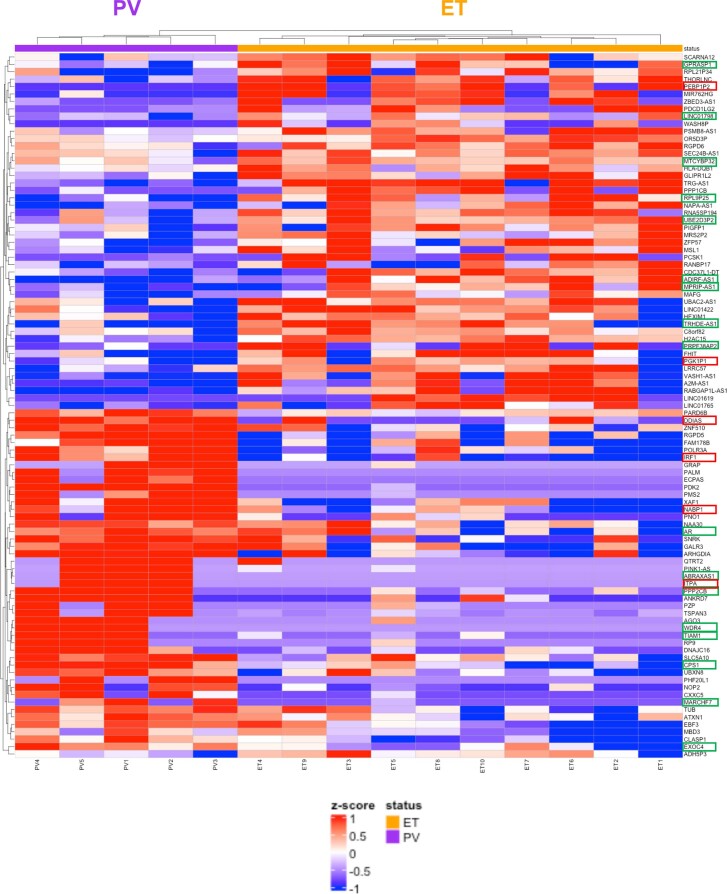
Gene set enrichment analysis of CDC42-interating proteins in human MPN. Top 100 featured genes in polycythemia vera (PV, n = 5) and essential thrombocythemia (ET, n = 10) from hHSC RNAseq dataset^45^. Genes in green boxes are related to activation and signalling downstream of CDC42, while genes in red boxes negatively correlate with CDC42 activity. Source data

**Extended Data Fig. 8:**
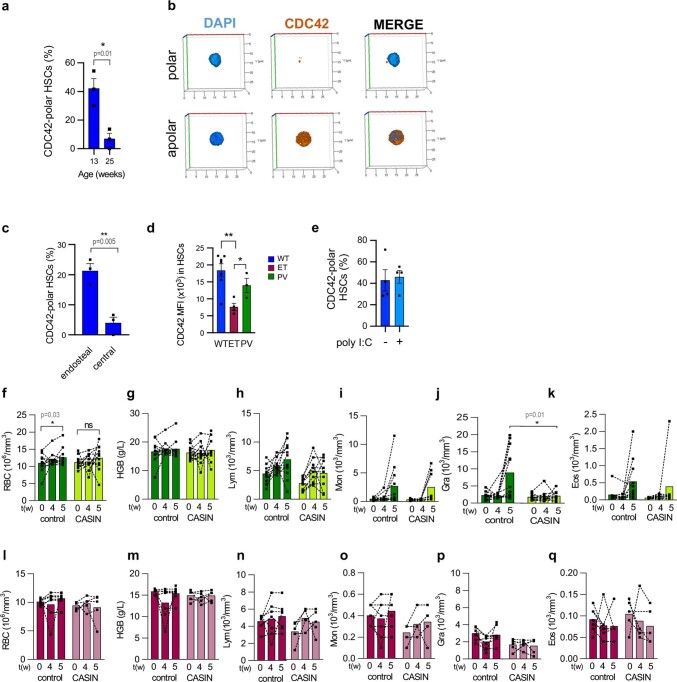
JAK-STAT signaling differentially regulates HSC CDC42-polarity in MPN subtypes. **a**, Frequencies of CDC42-polar HSCs in WT mice at different age (n = 3 mice). **b**, 3D representation showing the distribution of CDC42 (orange) and DAPI (blue) on polar or apolar HSCs imaged by confocal microscopy using the super resolution mode. **c**, Frequency of CDC42-polar HSCs isolated from the endosteal or central BM of WT mice 8w after transplantation of WT BM cells (n = 3 mice). **d**, CDC42 mean fluorescent intensity (MFI) in HSCs from 10-13w-old WT (n = 7 mice), ET-like (n = 4 mice) or PV-like (n = 3 mice) animals. **e**, Frequency of CDC42-polar HSCs isolated from 10– 13week-old, WT mice injected before (at 8w-old) with poly I:C (n = 4 mice) or vehicle (n = 4 mice). **f**-**q**, Blood counts before and 4-5w after CASIN treatment in PV-like (**f**-**k**) or ET-like (**l**-**q**) mice. **f**, **l**: Red blood cells (RBC). **g**, **m**, Hemoglobin (HGB). **h**, **n**, Lymphocytes (Lym). **i**, **o**, Monocytes (Mon). **j**, **p**, Granulocytes (Gra). **k**, **q**, Eosinophiles (Eos). **f**-**k**, Control, n = 11 mice; CASIN, n = 13 mice. **l**-**q**, Control, n = 7 mice; CASIN, n = 5 mice. **a**, **c**-**q**, Data are means ± SEM. Each square dot is a mouse. Two-sided Student’s t-test, *p < 0.05; **p < 0.01. Source data

To investigate the mechanism explaining the different niche location, we performed a supervised analysis of cell migration and polarity-related pathways in an hHSC RNA sequencing (RNA-seq) dataset^45^, which showed increased expression of gene sets related to activation and signaling downstream of the small Rho-GTPase CDC42 in PV hHSCs, while gene expression negatively correlated with CDC42 activity was comparatively enriched in ET hHSCs (Fig. 7a and Extended Data Fig. 7). CDC42 regulates HSC interactions with the niche^46^, HSC polarity, aging and myelopoiesis in mice^47^ and humans^48^. Additionally, reduced HSC polarity and increased CDC42 expression disrupt HSC interactions with endosteal BM niches during aging^47^. Therefore, we hypothesized that CDC42-mediated HSC polarity might underlie the differential interaction of HSCs with their BM niches in MPN. First, we measured polar HSCs and found that their frequency decreased already at mid-age (Extended Data Fig. 8a,b), which is consistent with progressive hematopoietic aging^49^. Endosteal HSCs were more polar compared with those in the central BM (Extended Data Fig. 8c). Matching the preferential endosteal location of ET-like HSCs, their CDC42 expression was reduced and their CDC42 polarity was increased in mid-aged mice (Fig. 7b,c and Extended Data Fig. 8d). Compared with ET-like HSCs, CDC42 expression was doubled in PV-like HSCs, which showed premature polarity loss (Fig. 7d); this was not a consequence of polyI:C administration to induce *Mx1*-Cre (Extended Data Fig. 8e). Treatment of HSCs with the CDC42 activity-specific inhibitor CASIN, which reportedly rejuvenates HSCs^47^, increased HSC polarity most pronouncedly in PV-like HSCs (Fig. 7c,d). In vivo, CASIN treatment for 3 days restored the endosteal location of PV-like HSCs measured through intravital microscopy (Fig. 7e,f). Similarly, chronic (5-week) CASIN treatment relocated PV-like HSCs to the endosteal BM, which is associated with increased frequency of quiescent HSCs (Fig. 7g–i), explaining fivefold-reduced BM HSCs (Fig. 7j). Consistently, CASIN treatment halved white blood cells (WBCs) and platelets (Fig. 7k,l and Extended Data Fig. 8f–k).

Chronic CASIN treatment of ET-like mice did not affect the (already) preferential endosteal location of HSCs (Fig. 7m); however, it similarly increased endosteal HSC quiescence, halving endosteal HSC numbers, without affecting HSCs, MPPs or overall blood counts (Fig. 7n–q and Extended Data Fig. 8l–q). These results suggest that differential CDC42 polarity in MPN HSCs regulates their localization and proliferation in different BM niches.

**Extended Data Fig. 9:**
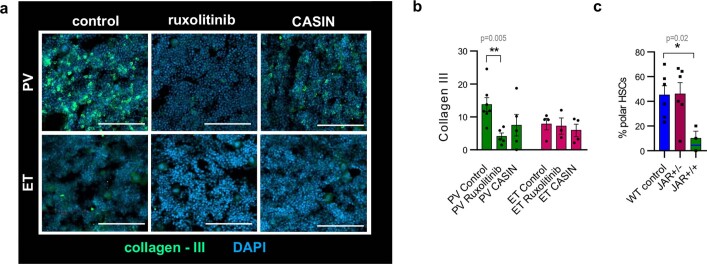
Increased collagen deposition in PV (compared with ET) model and CDC2-polarity loss dependent on JAK2^V617F^ expression. **a**, **b**, WT mice were lethally irradiated, transplanted with BM cells from PV-like or ET-like mice and treated with ruxolitinib (70 mg/kg, o.d., 3/w), CASIN (5 mg/kg/d, 3/w) or vehicle for 5w, starting 4w post-transplantation. **a**, Immunofluorescence of collagen III (green). Nuclei were counterstained with DAPI (blue). Scale bar, 100μm. **b**, Collagen III mean fluorescence intensity. Each square dot is a mouse. PV control, n = 7 mice; PV ruxolitinib, n = 5 mice; PV CASIN, n = 5 mice; ET control, n = 4 mice; ET ruxolitinib, n = 3 mice; ET CASIN, n = 4 mice. **c**, Frequency of CDC42-polar HSCs isolated from 10– 13w-old knock-in mice harboring one (*JAR*^*R/+*^) or two (*JAR*^*R/R*^) copies of the *JAK2*-V617F oncogene. WT, n = 6 mice; *JAR*^*R/+*^, n = 6 mice; *JAR*^*R/R*^, n = 3 mice. **b**, **c**, Data are means ± SEM. Two-sided Student’s t-test, *p < 0.05; **p < 0.01. Source data

Compared with ET, PV is associated with a higher transformation risk into secondary myelofibrosis^11,12^, which is characterized by increased BM collagen. This was recapitulated in MPN mice, with PV-like (but not ET-like) mice showing increased BM type III collagen content at the early disease stage; this was reverted by ruxolitinib and a similar trend was observed after CDC42 inhibition (Extended Data Fig. 9a,b). At more advanced MPN, secondary reticulin fibrosis in ET mice was improved by ruxolitinib and completely abrogated by CDC42 inhibition (Fig. 7r,s). Both treatments decreased osteoblast precursors, canceled osteosclerosis and reduced spleen size (Fig. 7t–w). These results suggest therapeutic effects of CDC42 inhibition in myelofibrosis.

### STAT1 and STAT5 differentially regulate HSC polarity

**Fig. 8:**
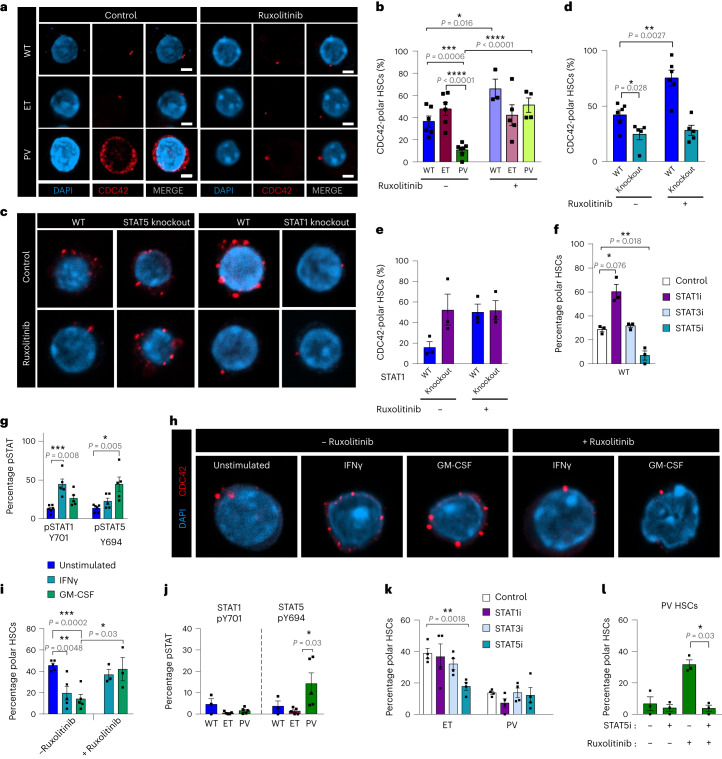
JAK–STAT signaling differentially regulates HSC polarity in MPN subtypes. **a**–**f**, CDC42 immunofluorescence (**a**,**c**, red) and frequencies of CDC42 polarity in LSK CD48^−^CD150^+^ HSCs (**b**–**f**). Scale bar, 2 µm. **b**, HSCs were sorted from WT (*n* = 3–6), ET-like (*n* = 5 or 6) or PV-like (*n* = 4–7) 10–13-week-old mice and cultured for 16 h with ruxolitinib (1 µM) or vehicle. *n* is the number of independent experiments. **d**, HSCs were sorted from 20-week-old STAT5 conditional knockout (*n* = 5) or WT (*n* = 5) mice. **e**, HSCs were sorted from 30-week-old STAT1 conditional knockout (*n* = 3) or WT (*n* = 3) mice. **f**, HSCs were sorted from 10–13-week-old WT mice and cultured for 16 h with STAT1 (NSC 118218 phosphate, 10 µM), STAT3 (BP-1-102, 5 µM) or STAT5 (AC-4-130, 5 µM) inhibitor, or vehicle (*n* = 3 mice). **g**, Frequency of pSTAT1^+^ (Y701) or pSTAT5^+^ (Y694) HSCs 15 min after in vitro stimulation with IFNγ (20 ng ml^−1^, *n* = 5 mice), GM-CSF (20 ng ml^−1^, *n* = 5 mice) or vehicle (*n* = 6 mice). **h**,**i**, CDC42 immunofluorescence (**h**, red) and frequencies of CDC42-polar HSCs (*n* = 3–5 mice) (**i**). Fluorescence-activated cell-sorted HSCs were cultured for 16 h with IFNγ (20 ng ml^−1^) or GM-CSF (20 ng ml^−1^), with or without ruxolitinib (1 µM), and stained for CDC42. **j**, Frequency of pSTAT1^+^ (Y701) or pSTAT5^+^ (Y694) HSCs isolated from WT (*n* = 3), ET-like (*n* = 5) or PV-like (*n* = 5) mice. **k**, Frequencies of CDC42-polar HSCs from 10–13-week-old ET-like or PV-like mice after 16-h culture with STAT1 (NSC 118218 phosphate, 10 µM), STAT3 (BP-1-102, 5 µM) or STAT5 (AC-4-130, 5 µM) inhibitors, or vehicle (*n* = 4 mice). **l**, Frequencies of CDC42-polar HSCs from PV-like mice after 16-h culture with vehicle, STAT5 inhibitor (AC-4-130, 5 µM), ruxolitinib (1 µM) or a combination of both (*n* = 3 mice). In **b**,**d**–**f**,**g**,**i**–**l** Data are shown as the mean ± s.e.m. Each square dot is a mouse. *P < 0.05, **P < 0.01, ***P < 0.001, ****P < 0.0001. In **b** a two-sided one-way ANOVA was used. In **d**–**f**,**g**,**i**–**l** a Student’s *t*-test was used. Source data

**Extended Data Fig. 10:**
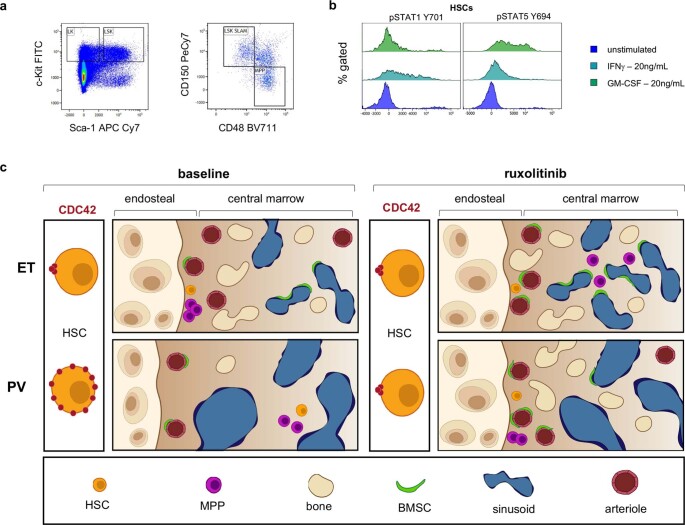
Model of different niches for mutant stem cells affecting pathogenesis and therapy response in MPN. **a**, Flow cytometry showing the gating strategy and preserved immunophenotype of Lin^−^Sca1^+^cKit^+^ (LSK) CD48^−^CD150^+^ HSCs after fixation and permeabilization, which allowed reliable quantification of phosphorylated (p) STAT proteins. **b**, Representative flow cytometry diagram showing the expression of pSTAT1 or pSTAT5 in HSCs after exposure to IFNγ or GM-CSF. **c**, Model of different niches for mutant stem cells affecting pathogenesis and therapy response in MPN. Essential thrombocythemia (ET) and polycythemia vera (PV) hematopoietic stem cells (HSCs) exhibit opposite JAK-STAT dependent alterations in the distribution of the small Rho-GTPase CDC42. PV HSCs become apolar prematurely, while ET HSCs retain high polarity, leading to different lodgment, proliferation and microenvironmental remodeling in central sinusoidal or endosteal BM niches, respectively. Endosteal arterioles and bone increase in ET, while PV causes sinusoidal vasodilatation. The JAK2 inhibitor ruxolitinib restores endosteal the PV-HSC niche but relocates multipotent progenitors (MPP) to the central BM in ET, which possibly explains the variable clinical response to ruxolitinib in these MPN subtypes. Source data

Altered CDC42 expression in *JAK2*^V617F+^ HSCs suggested that JAK2 might regulate their CDC42 polarity. Indeed, premature CDC42 polarity loss was recapitulated in *JAK2*^V617F^ knockin mice^33^ but only detected in HSCs with two mutant alleles (Extended Data Fig. 9c). Correspondingly, treatment with ruxolitinib increased WT or PV-like HSC polarity (Fig. 8a,b), resembling the effects of CASIN (Fig. 7b–d). Ruxolitinib’s effect was STAT-dependent, as ruxolitinib did not rescue CDC42 polarity in STAT5- or STAT1-deficient HSCs (Fig. 8c–e). However, the baseline frequencies of polar HSCs were inverted in both knockout mice, suggesting a different impact of STAT1 and STAT5 on CDC42 polarity. This was confirmed using STAT1/5 inhibitors, while STAT3 inhibition did not affect WT HSC polarity (Fig. 8f). These results suggest that STAT1 may hinder, but STAT5 preserves, WT HSC CDC2 polarity. Unphosphorylated and phosphorylated STAT differentially regulate gene expression^50^ (see Yang and Stark^51^ for a review). To separate both, we tested cytokines triggering STAT phosphorylation and found that interferon-γ (IFNγ) increases pSTAT1, while granulocyte-macrophage colony-stimulating factor (GM-CSF) induces pSTAT5 in WT HSCs (Fig. 8g and Extended Data Fig. 10a,b). IFNγ or GM-CSF reduced WT HSC CDC42 polarity, which was reversed by ruxolitinib (Fig. 8h,i), implying that pSTAT1 and pSTAT5 decrease HSC polarity. Together, these data suggest that unphosphorylated and pSTAT1 decrease HSC CDC42 polarity, while STAT5 might have a dual function depending on its phosphorylation (pSTAT5 decreases but unphosphorylated STAT5 maintains CDC42 polarity in WT HSCs).

### JAK–STAT signaling regulates HSC polarity in MPN subtypes

Finally, we asked whether the different CDC42 polarity in ET-like and PV-like HSCs may be explained by distinct JAK–STAT signaling. The baseline frequencies of pSTAT5^+^ (not pSTAT1^+^) HSCs were higher in PV-like than in ET-like or WT mice (Fig. 8j). Unlike STAT1 inhibition, STAT5 inhibition (blocking both unphosphorylated and pSTAT5) halved the frequency of ET-polar HSCs but it did not affect the (already low) polarity of PV-like HSCs (Fig. 8k). This suggests that increased pSTAT5 in PV HSCs reduces CDC42 polarity, while unphosphorylated STAT5 might preserve CDC42 polarity in WT and ET HSCs. Indeed, STAT5 inhibition suppressed the rescue of PV-like HSC polarity by ruxolitinib, suggesting that ruxolitinib increases HSC CDC42 polarity, endosteal lodgment and quiescence through unphosphorylated STAT5 signaling (Fig. 8l).

Overall, these results indicate that heterogeneous JAK–STAT signaling regulates CDC42-dependent HSC polarity and niche interactions in MPN subtypes, leading to differential remodeling of the microenvironment and response to JAK inhibitor (Extended Data Fig. 10c).

## Discussion

In this study, we addressed the question whether the stem cell niche influences the growth of cancer stem cells affected by the same oncogenic pathway and thereby explain different disease development and therapy response. We have used *JAK2*^V617F^ as one of the most common mutations causing CH^1^ and giving rise to different MPN subtypes with distinct progression^2–5^ and response to JAK inhibitors^13–15^. The evaluation of clinical samples, different transgenic and knockin mice, and PDX models carrying hHSCs from PV and ET unravel important differences in the histopathological features of MPN subtypes, provide insights into MPN pathogenesis and help to explain the variable response to JAK inhibition.

The results indicate that *JAK2*^V617F^-mutated HSCs preferentially occupy and remodel different BM niches in ET and PV. Human and mouse HSPCs are found in contact to endosteal arterioles and capillaries in ET, whereas a perisinusoidal hHSPC location appears more frequently in human PV. These heterogeneous HSC-niche interactions are confirmed through longitudinal in vivo imaging studies and result from the variable impact of JAK–STAT signaling on HSC-niche interactions in MPN subtypes.

Different *JAK2*^V617F^ expression and thresholds are required to activate erythropoietin and thrombopoietin receptors, possibly explaining the specific phenotypes (erythrocytosis and thrombocythemia) in PV and ET, respectively^52^. Although advanced disease can cause myelofibrosis and osteosclerosis in all MPN subtypes^11,12^, our results indicate that the interaction of mutant HSCs with endosteal or central BM niches contributes to explain the different aggressiveness of PV and ET, despite their shared oncogenic driver. This is suggested by lower overall mutant ET cell expansion in the endosteum, compared with increased PV cell proliferation in central BM niches. The endosteal niche is rich in arterioles and capillaries, and promotes HSC quiescence^17,20,22,39–42^, while active HSCs transmigrate through sinusoids, which are abundant in central BM^24^. Reduced endosteal and increased central BM niches promote myeloid cell expansion during aging^28,29^. These features are found prematurely in the BM microenvironment lacking β_3_-AR^26,28^, which accelerates ET development due to increased proliferation of megakaryocyte-committed cells in the central BM. Therefore, microenvironmental aging might increase MPN risk.

The reason for distinct HSC-niche interactions in MPN subtypes appears to be opposite alterations in the small Rho-GTPase CDC42, which regulates HSC interactions within the niche^46^, HSC polarity, aging and myelopoiesis in mice^47^ and humans^48^. CDC42 deficiency dislodges normal HSCs from the ‘restrictive’ endosteal niche and relocates HSCs toward the central BM, where these cells find a more ‘permissive’ microenvironment for proliferation^53^ and myeloid differentiation^54^. Furthermore, CDC42 mutations have been associated with human infantile myeloproliferation^55^. We found that ET-like HSCs retained low CDC42 expression and high polarity in mid-age, while PV-like HSCs exhibited premature polarity loss. Treatment of HSCs with the CDC42 activity-specific inhibitor (CASIN), which reportedly rejuvenates HSCs^47^, restores PV-like HSC polarity and quiescence in the endosteal niche, improving circulating leukocytes and platelets. Contrastingly, CDC42 inhibition does not affect the endosteal HSC location in ET, or overall blood counts. Therefore, these results suggest that differential CDC42 polarity in MPN HSCs regulates their localization and proliferation in different BM niches.

JAK–STAT signaling regulates CDC42 during monocyte migration^56^. Therefore, we hypothesized that heterogeneous STAT signaling in MPN subtypes differentially regulates CDC42 polarity. Indeed, genetic or pharmacological STAT1 and STAT5 loss of function oppositely alter polar HSC frequency. STAT1 deletion increases, but STAT5 deletion decreases HSC CDC42 polarity. However, phosphorylation of STAT1 and STAT5 similarly reduces HSC CDC42 polarity. This suggests opposite effects of STAT5 on CDC42 polarity dependent on phosphorylation, which affects its gene regulatory function^50,51^. Interestingly, CDC42 effector proteins 1, 2 and 5 were found among the top 40 upregulated genes after STAT5 knockdown in the mHSC-like HPC-7 cell line^50^, further suggesting that the balance between unphosphorylated and pSTAT5 regulates HSC CDC42 polarity and niche interactions. In our study, ruxolitinib increased WT and PV HSC CDC42 polarity via STAT5. Together, our results point toward increased pSTAT5 in PV HSCs as the probable cause of premature polarity loss and disengagement from the endosteal niche, which promotes HSC quiescence.

Ruxolitinib is superior to second-line treatments in PV^14,15^ but not in ET^13^; however, the underlying reasons for this discrepancy are unknown. Our results provide some cues by uncovering important differences in ruxolitinib’s effects on MPN subtypes, possibly explaining the variable clinical response. Ruxolitinib treatment triggers endosteal lodgment and quiescence of HSCs and MPPs, arteriole and capillary and endosteal niche expansion in PV-like mice. Similarly, 1-year treatment with ruxolitinib (but not with BAT) doubles BM arterioles and capillaries and bone area, and hHSPCs become closer to arterioles and capillaries or bone in human PV. Contrastingly, ruxolitinib treatment of ET-like mice does not affect the endosteal HSC location but relocates MPPs to the central BM, causing central sinusoidal vasodilation and endosteal niche constriction, and overall HSPC expansion. Similarly, sinusoid increase and HSPCs move closer to them in ruxolitinib-treated patients with ET. Overall, many uncovered histopathological and HSPC-niche features of ET and PV are swapped by ruxolinitib treatment. Therefore, these results suggest that dissimilar HSC-niche interactions underlie a distinctive response to JAK inhibitor, possibly explaining the more pronounced therapeutic effects of ruxolitinib in patients with PV (compared with patients with ET)^13–15^.

Altogether, these results illustrate how cancer stem cells carrying the same oncogenic driver can generate diseases with different penetrance and response to therapy dependent on specific interactions with their niches. Therefore, interfering with stem cell niche interactions might impact disease progression in MPNs and possibly in other premalignant disorders.

## Methods

### Human studies

All centers had appropriate research and ethical approval; patients gave their written informed consent. Some samples were derived from the Cambridge Biobank (18/EE/0199) and patients attending outpatient clinics at Addenbrooke’s Hospital (UK), under the clauses of the Causes of Clonal Haematological Disorders project, which had regional ethical approval from the Eastern Multi-region Ethics Committee (MREC 02/5/22 and 07/MRE05/44) and local research and ethical approval at participating UK hospitals. Other samples were derived from patients enrolled in the MAJIC Phase-2 clinical study (ISRCTN61925716)^13^. For xenotransplantation, BM or peripheral blood was collected from three patients with ET and three patients with PV after obtaining informed consent, under studies approved by the local ethics committee (KEK-ZH-NR: 2009-0062/1 and BASEC-NR: 2018-00539). Both male and female patients were included in the different clinical studies. Differences regarding sex were not investigated in this study. The information on the disaggregation between sex and gender was not collected.

### Mouse strains

Mice were housed in specific pathogen-free facilities. All experiments using mice followed protocols approved by the Animal Welfare Ethical Committee, according to the United Kingdom Home Office regulations (PPL P0242B783 and PPL 708403) or in accordance with the Swiss Federal Veterinary office and the cantonal veterinary office of Zurich, Switzerland. Mice were housed in specific pathogen-free facilities in individually ventilated cages under 12 h light–dark cycles and controlled temperature (19–23 °C) and humidity (55 ± 10%) with free access to standard rodent chow (SafeDiet R105-25). Mice were housed in individually ventilated cages, all diet was irradiated and cages, bedding and environmental enrichment were autoclaved. Full cage changes were performed in changing stations and any procedures were carried out in a CLII cabinet. The Health Monitoring Surveillance Program consisted of the microbiology analysis of mouse sentinels and contact animals according to the Federation of European Laboratory Animal Science Associations (FELASA) recommendations. Every quarterly period, sentinels and contact animals of the rack were bled for serology and tested for the agents recommended. At Cambridge University, FELASA PLUS screening was performed annually and *Klebsiella* spp. were analyzed as an additional agent. The humane endpoint was defined by the project license in accordance with the Home Office regulations as a 15% loss of maximal body weight; all mice were euthanized before or on reaching this stage. *Nes-GFP*^57^, FVB/N-*Adrb3*^*tm1Lowl*^/J (stock no. 006402, The Jackson Laboratory), B6.129S(Cg)-*Stat1*^*tm1Dlv*^/J (stock no. 012606, The Jackson Laboratory), B6.129S6-*Stat5b*^*t*^^*m1Mam*^ *Stat5*^*atm2Mam*^/Mmjax (stock no. 032053, The Jackson Laboratory), B6.FVB-Tg(Acta2-DsRed)1Rkl/J (stock no. 031159, The Jackson Laboratory), VWF-TdTomato^44^, *Vav-Cre;JAK2*^[V617F30^, *Mx1-Cre;JAK2*^V617F^ (ref. ^30^), *JAK2*^[V617F33^, *Scl-tTA;JAK2*^V617F^ (ref. ^31^), CALR^del/+REF38^, MISTRG mice^35^ and congenic B6.SJL-*Ptprc*^*a*^*Pepc*^*b*^/BoyJ (CD45.1), CD45.2 C57BL/6 mice (Charles River Laboratories) were used in this study. *Vav-Cre;JAK2*^V617F^ mice were used as the ET-like model. *Vav-Cre;JAK2*^V617F^ mice express active Cre in fetal and adult HSCs. *Mx1-Cre;JAK2*^V617F^ (ref. ^30^), *JAK2*^R/R^ (ref. ^33^) and *Scl-tTA;JAK2*^V617F^ (ref. ^31^) mice were used as independent PV-like models. In double transgenic *Mx1-Cre;JAK2*^V617F^ mice, Cre activation was induced through intraperitoneal injection of polyinosinic–polycytidylic acid sodium salt (catalog no. P1530, Sigma-Aldrich). In *Scl-tTA;JAK2*^V617F^ mice, *JAK2*^V617F^ expression was induced by intraperitoneal injection of 143 mg kg^−1^ tamoxifen (three times on alternate days)^31^. Both female and male mice were used in the different experiments; possible differences regarding sex were not investigated in this study. Mice with similar blood counts were randomly allocated to control or treatment groups.

### BM transplantation

Age-matched, CD45.1 or CD45.2 C57BL/6J mice (8–12 weeks old) were used as recipients in the BM transplantation assays. Recipients were subjected to lethal irradiation (12 Gy whole-body irradiation, split dose 6.0 + 6.0 Gy, 3 h apart) before injection. For competitive transplantation, 10^6^ BM cells from a *Mx1-Cre;JAK2*^V617F^*;*DsRed^+^ CD45.2 donor and 10^6^ BM cells from a *Vav-Cre;JAK2*^V617F^ CD45.2 donor were injected into the tail vein of CD45.1 mice. For noncompetitive transplant, 2 × 10^6^ BM cells were injected.

### In vivo treatments

CASIN (catalog no. 5050, Tocris Biosciences) powder was resuspended in dimethyl sulfoxide (DMSO) at 100 mM, stored at −20 °C, thawed and diluted in PBS before injection. Ruxolitinib (Jakavi, Novartis) was resuspended in polyethylene glycol (catalog no. 202371-500g, Sigma-Aldrich) for administration to mice. Mice were treated with CASIN (5 mg kg^−1^, intraperitoneally), ruxolitinib (70 mg kg^−1^, once daily) or vehicle every other day for 5 weeks. For intravital imaging, 8–12-week-old *Nes-GFP* recipient mice were irradiated and transplanted with 1,000–5,000 Lin^−^CD150^+^CD48^−^ HSCs isolated from β-actin-DsRed donors (B6.FVB-Tg(Acta2-DsRed)1Rkl/J mice intercrossed with *Vav-Cre;JAK2*^V617F^ or *Mx1-Cre;JAK2*^V617F^ mice). Recipient mice were treated with CASIN (10 mg kg^−1^, intraperitoneally), ruxolitinib (70 mg kg^−1^, once daily) or vehicle 24, 48 and 72 h after transplantation. The last treatment was performed 2 h before the beginning of the surgery for intravital imaging.

### Intravital microscopy

Intravital microscopy was performed as described before^58^ using a ZEISS LSM 780 upright confocal microscope with a motorized stage and the following lasers: Argon, 561, 633 and a tunable infrared multiphoton laser (Spectraphysics Mai Tai DeepSee 690-1040). Signal was visualized with a W Plan-Apochromat ×20 DIC water immersion lens (1.0 numerical aperture). Anesthesia was induced in mice with 4% isoflurane mixed with pure oxygen. This was gradually reduced to approximately 1% as anesthesia stabilized. To ensure steady positioning of mice on the microscope, surgery to attach the headpiece and imaging window was then performed as described by Scott et al.^58^. Large three-dimensional (3D) tile scans of the entire BM cavity space were acquired by stitching adjacent, high-resolution Z-stack images. Blood vessels were highlighted by intravenous injection of 50 μl of 8 mg ml^−1^ 500 kDa Cy5-Dextran (Nanocs). For repeated imaging, protective intrasite gel (Smith & Nephew) was applied to the imaging window to preserve bone integrity and prevent scar formation. The window was bandaged and mice were allowed to recover from anesthesia. Owing to the lock-and-key mechanism of the imaging window^58^, mice could then be re-anesthetized and accurately repositioned on the microscope stage and the same BM areas reimaged. After each imaging session, analgesia was administered via oral buprenorphine in raspberry jelly at a dose of approximately 0.8 mg kg^−1^. Imaging sessions were performed on days 1 and 3 after injection of the cells on day 0. Altogether, 21 images were collected during each imaging session. Stills from intravital imaging were analyzed with the Volocity software (PerkinElmer) to measure in 3D space the distances between the DsRed-labeled cells and the bone surface. The minimal distance module within the Volocity software was used. Positions XYZ for every cell were analyzed as described by Scott et al.^58^.

### Xenograft

Primary hHSPCs (CD34^+^) cells were purified from the BM or peripheral blood of patients using Ficoll density gradient centrifugation and further magnetically isolated using the MACS CD34 MicroBead Kit (Miltenyi Biotec). CD34^+^ cells were cryopreserved and slowly thawed in IMDM with 50% FCS at 37 °C before xenotransplantation and resuspended in 25 µl PBS for injection. MISTRG mice aged 8–12 weeks old^59^ were irradiated sub-lethally (181 cGy using an X-ray RS-2000 irradiator, Rad Source) and transplanted with a 22-gauge needle (Hamilton Company) intrafemorally with 1.5–3 × 10^5^ CD34^+^ hHSPCs.

### Immunohistochemistry of human BM trephines

A conventional immunohistochemistry protocol was performed on paraffin sections. Briefly, sections were deparaffinized in xylene followed by progressive rehydration with decreasing concentrations of ethanol. Antigen retrieval was performed by proteolytic enzyme digestion using Tris-EDTA Buffer (10 mM Tris; 1 mM EDTA, 20 mg ml^−1^ proteinase K, pH 8). Endogenous peroxidase was quenched using H_2_O_2_ 30% in TBS 1×, before being blocked with 10% goat serum, 10 mg ml^−1^ BSA, 0.1% Triton X-100, TBS. Sections were incubated with avidin and biotin (Vector Laboratories), anti-CD34 antibody (catalog no. MA1-10202, Thermo Fisher Scientific) and horseradish peroxidase*-*coupled secondary antibody (catalog no. 115-035-006, The Jackson Laboratory). Samples were blocked with the ABC Kit (Vector Laboratories) and incubated with a substrate of peroxidase, 3,3′-diaminobenzidine (SIGMAFAST, Sigma-Aldrich). Sections were stained in hematoxylin, rinsed in water and dipped in 1% acid alcohol, and then mounted with aqueous mounting medium (Vector Laboratories). Slides were imaged using a slide scanning microscope (ZEISS Axioscan) and analyzed manually to measure the distance and areas, and quantify cells and vessels using the NDP2.view software (Hamamatsu).

### Histology of mouse bones

Femurs and tibias were collected, cleaned and put in PBS, 2% paraformaldehyde (PFA) (Sigma-Aldrich) overnight. For cryosectioning and immunostaining, bones were washed once with PBS, decalcified in 250 mM EDTA and PBS for 1.5 weeks at 4 °C, put in 15% sucrose and PBS for 24 h then in 30% sucrose and PBS for another 24 h, and embedded with OCT (catalog no. 12678646, Thermo Fisher Scientific) in plastic cryomolds (catalog no. 4557, Sakura), using chilled methyl-butane (Sigma-Aldrich) for snap-freezing. The samples were stored at −80 °C and sections (12 µm) or whole mount were obtained using a cryostat (Leica Biosystems). Alternatively, after fixation bones were embedded in paraffin and 5-µm-thick sections were stained with hematoxylin and eosin and Masson’s trichome for conventional morphological evaluation. The Acid Phosphatase Leukocyte Kit (TRAP, catalog no. 387A, Sigma-Aldrich) staining was used according to the manufacturer’s recommendations.

### Immunofluorescence of cryosections

Immunofluorescence staining of cryosections or whole mounts was performed as described previously^60^ using femurs, with minor modifications. Briefly, samples were permeabilized with 0.1% Triton X-100 (Sigma-Aldrich) in Tris-NaCl-blocking (TNB) buffer (0.1 M Tris-HCl, pH 7.5, 0.15 M NaCl, 0.5% blocking reagent, PerkinElmer) at 4 °C. Samples were incubated with primary antibodies: anti-CD31 (1:100, catalog no. AF3628, R&D Systems); anti-EMCN (1:100, catalog no. sc-65495, Insight Biotechnology); anti-Sp7 (1:200, catalog no. ab22552, Abcam); anti-CD31 (1:200, Clone MEC13.3, BD Biosciences); anti-OPN (1:100, catalog no. AF808, R&D Systems); anti-CD117 (1:200, catalog no. AF1356, R&D Systems); anti-Ki67 (1:100, catalog no. ab15580, Abcam); anti-Collagen III (1:100, catalog no. PA5-34787, Thermo Fisher Scientific), diluted in 0.1% Triton X-100 TNB for 3 days on horizontal shaking (whole mount) or O/N (cryosection) at 4 °C. Samples were rinsed with PBS four to five times for 24 h (whole mount) or 10 min (cryosection) and incubated for 24 h (whole mount) or 1 h (cryosection) with secondary antibody diluted at 1:300 in TNB: donkey anti-goat AF488 (catalog no. A11055, Thermo Fisher Scientific); donkey anti-rabbit AF488 (catalog no. A21206, Thermo Fisher Scientific); donkey anti-rat AF488 (catalog no. A21208, Thermo Fisher Scientific); donkey anti-goat AF546 (catalog no. A11056, Thermo Fisher Scientific); donkey anti-rabbit AF546 (catalog no. A10040, Thermo Fisher Scientific); donkey anti-rat AF555 (catalog no. A21434, Thermo Fisher Scientific); donkey anti-goat AF647 (catalog no. A21447, Thermo Fisher Scientific); donkey anti-rabbit AF647 (catalog no. A31573, Thermo Fisher Scientific); and donkey anti-rat DyLight 650 (catalog no. SA5-10029, Thermo Fisher Scientific). Repetitive washes were performed with PBS for 1 day (whole mount) or 5 min twice (cryosection). Stained tissue sections were counterstained for 10 min with 5 mM DAPI in PBS and rinsed with PBS. For sections, slides were mounted in mounting medium (catalog no. S3023, DAKO). Images were acquired with a confocal microscope (Leica SP5, Leica SP8, Stellaris or ZEISS 980) using 10*×*, 20*×* and 40*×* objectives and analyzed with ImageJ. At least two independent and randomly selected BM areas in the diaphysis were imaged and analyzed per sample. Arterioles (small caliper, CD31^hi^EMCN^−^ vessel) number was counted and normalized to the BM area. For the quantification of sinusoid diameter, the diameter of 20 random sinusoids was measured and the average was calculated for each sample. The quantification of the frequency of different cell populations using DAPI, CD117, Ki67 and VWF staining was performed using ImageJ and the CellProfiler software. The endosteal area of the sample was defined as the area within 150 µm from the bone surface; the central BM was considered as the area localized more than 150 µm away from the bone surface.

### RNA isolation and quantitative PCR

RNA isolation was performed using TRIzol Reagent (catalog no. T9424, Sigma-Aldrich) on mouse whole BM cells. Reverse transcription was performed using the High-Capacity cDNA Reverse Transcription Kit (catalog no. 4368814, Applied Biosystems) according to the manufacturer’s recommendations. Quantitative PCR was performed using the PowerUp SYBR Green Master Mix (catalog no. A25742, Applied Biosystems) and ABI PRISM 7900HT Sequence Detection System. The expression level of each gene was determined by using the absolute quantification standard curve method. All values were normalized with *Gapdh* as the endogenous housekeeping gene.

The following primers (Sigma-Aldrich) were used: *Col1a1*-forward: TATTGCTGGACAACGTGGTG; *Col1a1*-reverse: ACCTTGTTTGCCAGGTTCAC; *Gapdh*-forward: GCATGGCCTTCCGTGTTC; *Gapdh*-reverse: CTGCTTCACCACCTTCTTGAT.

### Immunofluorescence of sorted HSCs

*Mx1-Cre;JAK2*^V617F^, *Vav-Cre;JAK2*^V617F^ or Cre-negative control mice were euthanized, BM cells were immunomagnetically depleted of hematopoietic lineage marker-expressing cells using biotin-conjugated lineage cocktail, magnetic streptavidin-conjugated beads (catalog no. 557812, BD Biosciences) and cell separation magnet (catalog no. 552311, BD Biosciences) according to the manufacturer’s recommendations. Chambered coverslips (catalog no. 81811, Ibidi) were coated with fibronectin (40 µg ml^−1^) (F1141-1mg, Sigma-Aldrich) in NaHCO_3_ overnight at 4 °C and washed with PBS. Lin^−^Sca1^+^cKit^+^CD150^+^CD48^−^DAPI^−^ HSCs were sorted and seeded on the coverslips in IMDM (catalog no. 21056-023, Thermo Fisher Scientific) supplemented with 10% FCS, 1% penicillin-streptomycin, and treated with ruxolitinib (INCB018424, resuspended in DMSO, final concentration 1 µM), CASIN (1 µM, TOCRIS), AC-4-130 (5 µM, STAT5 inhibitor, MedChemExpress), NSC 118218 phosphate (10 µM, STAT1 inhibitor, MedChemExpress), BP-1-102 (5 µM, STAT3 inhibitor, Selleck Chemicals) or DMSO for 16 h at 37 °C, 5% CO_2_. The medium was carefully removed and cells were fixed with BD Cytofix Fixation Buffer (catalog no. BD 554655, BD Biosciences) for 20 min at room temperature. Cells were washed gently with PBS and permeabilized with 0.2% Triton X-100 (catalog no. 9002-93, Sigma-Aldrich) in PBS for 20 min, washed and blocked for 1 h with 20% donkey serum (catalog no. D9663, Merck) in PBS. Cells were incubated with the primary antibody (rabbit anti-mouse CDC42, 1:100, catalog no. 07-1466, Merck) in 5% donkey serum O/N at 4 °C, and washed twice with PBS before incubation with secondary antibody (donkey anti-rabbit AF647, 1:300, catalog no. A-31573, Thermo Fisher Scientific) for 1 h at room temperature and washed twice with PBS. Cells were stained with DAPI for 2 min at room temperature, washed and mounted using fluorescence mounting medium (catalog no. S3023, DAKO). Imaging was performed at 40× or 63× using the super resolution mode with the Airyscan on a ZEISS 980 confocal microscope. To define the polarity, the distribution of CDC42 was analyzed across the whole Z-stack and the HSC was considered polar when a clear asymmetric distribution of the protein was visible. A minimum of 20 HSCs per condition were analyzed. The 3D reconstitution of the HSC was obtained using the Imaris software.

### µCT

Femurs were collected and the attached soft tissue was removed thoroughly and fixed in 4% PFA. The fixed femurs were scanned using a SkyScan 1174 scanner: 50 kV, 800 μA, 8.3-μm isometric voxel resolution and 0.7-degree rotation step. Images were analyzed using the SkyScan CT Analyzer software v.1.9.3.0.

### BM cell extraction, flow cytometry and cell sorting

Hematopoietic cell isolation from BM or PB was performed as described previously^28^. The marrow was flushed and the bones crushed with a mortar and pestle (catalog no. 10656405, Fisher), in PBS, 2% FCS and filtered through a 40-µm strainer (catalog no. 542040, Greiner Bio-One). PB or BM cell suspension was depleted of red blood cells using the ACK lysis buffer for 8 min at 4 °C (catalog no. 420301, BioLegend), washed and numerated using trypan blue (catalog no. 10593524, Fisher). Cells were incubated with the appropriate dilution (2–5 mg ml^−1^) of fluorescent antibody conjugates. DAPI (catalog no. D9542, Sigma-Aldrich) or 7-AAD (catalog no. 420404, BioLegend) was added to discriminate dead cells. Samples were analyzed with an LSRFortessa flow cytometer (BD Biosciences) or sorted (FACSAria, BD Biosciences) equipped with the FACSDiva Software (BD Biosciences). The following antibodies were used to detect the human hematopoietic cells in the PDX model: PE-conjugated mouse anti-human CD45 (catalog no. 555483, BD Biosciences); APC-conjugated mouse anti-human CD34 (catalog no. 555824, BD Biosciences); and PE-Cy7-conjugated mouse anti-human CD38 (catalog no. 560677, BD Biosciences). The following antibodies were used for the staining of the mouse hematopoietic cells in different panels: biotin-conjugated lineage cocktail (catalog no. 133307, BioLegend); APC-Cy7-conjugated anti-Sca1 (catalog no. 108126, BioLegend); FITC-conjugated anti-CD117 (catalog no. 105805, BioLegend); BV711-conjugated anti-CD48 (catalog no. 103439, BioLegend); PE-Cy7-conjugated CD150 (catalog no. 115914, BioLegend); PE-conjugated streptavidin (catalog no. 405207, BioLegend); APC-Cy7-conjugated anti-CD117 (catalog no. 105826, BioLegend); BV421-conjugated anti-Sca1 (catalog no. 108128, BioLegend); BV605-conjugated anti-CD150 (catalog no. 115927, BioLegend); PE-CY7-conjugated anti-CD117 (BioLegend, cat. no. 105814, BioLegend); APC-Cy7-conjugated anti-CD45.2 (catalog no. 25-0454-U100, Insight); BV605-conjugated anti-CD41 (catalog no. 133921, BioLegend); BV711-conjugated anti-CD16/CD32 (catalog no. 101337, BioLegend); APC-conjugated anti-CD71 (catalog no. 17-0711-80, Thermo Fisher Scientific); BV605-conjugated anti-Ter119 (catalog no. 116239, BioLegend); FITC-conjugated anti-CD34 (catalog no. 553733, BD Biosciences); biotin-conjugated anti-CD3e (catalog no. 553060, BD Biosciences); PE-conjugated anti-Ly6G (catalog no. 108408, BioLegend); BV421-conjugated anti-CD11b (catalog no. 101235, BioLegend); BV510-conjugated streptavidin (catalog no. 405234, BioLegend); AF488-conjugated streptavidin (catalog no. 405235, Invitrogen). For the erythroprogenitor analysis, we followed the same protocol but did not perform the red blood cell lysis buffer.

For the cell cycle analysis, cells were stained as described above, permeabilized with the Cytofix/Cytoperm Kit (catalog no. BDB554714, BD Biosciences), stained with the APC-conjugated anti-Ki67 antibody (catalog no. 652405, BioLegend) O/N at 4 °C in Perm/Wash buffer (catalog no. BD554723, BD Biosciences), stained with Hoechst 33342 (catalog no. 62249, Thermo Fischer Scientific) and analyzed. Sub-G0 cells were excluded and the percentage of cells in the different phase was calculated among the G0, G1, S, G2 and M phases. For CDC42 total protein analysis by flow cytometry, cells were stained for the membrane panel as described above and permeabilized with the Cytofix/Cytoperm kit (catalog no. BDB554714, BD Biosciences), stained with the anti-CDC42 antibody in Perm/Wash buffer 5% donkey serum O/N at 4 °C, washed and stained with donkey anti-rabbit AF647 in Perm/Wash buffer, washed and analyzed. For stromal cell analysis, BM was flushed and the remaining bones were crushed in PBS and digested in collagenase (catalog no. 07902, Stem Cell Technologies) for 30 min at 37 °C in a water bath with agitation. The enzyme was quenched by adding PBS with 2% FCS. Cell suspensions were filtered, pelleted and red blood cell lysis was performed as stated above. Samples were stained with the following antibodies: biotin-conjugated anti-Ter119 (catalog no. 553672, BD Biosciences); biotin-conjugated anti-CD45 (catalog no. 553078, BD Biosciences); PE-Cy7-conjugated anti-CD31 (catalog no. 102524, BioLegend); PE-conjugated anti-EMCN (catalog no. sc-665495 PE, Santa Cruz Biotechnology); APC-Cy7-conjugated anti-Sca1 (catalog no. 108126, BioLegend); BV421-conjugated rat anti-mouse CD51 (catalog no. 740062, BD Biosciences); APC-conjugated anti-CD140α (catalog no. 135908, BioLegend); BV710-conjugated streptavidin (catalog no. 405241, BioLegend).

### Steady-state STAT signaling analysis in mHSCs

Age-matched *Vav-Cre;JAK2*^V617F^*, Mx1-Cre;JAK2*^V617F^ or control mice were culled by cervical dislocation and the bones were crushed soon after death with Phosflow Lyse/Fix Buffer (catalog no. 558049, BD Biosciences) according to the guidelines from the supplier. Cells were washed using Perm/Wash buffer and stained the LSK SLAM panel as described above and incubated O/N at 4 °C with an anti-pSTAT1 (catalog no. 612597, BD Biosciences), an anti-pSTAT5 (catalog no. 612599, BD Biosciences) or isotype control (catalog no. 557783, BD Biosciences), then washed and analyzed on an LSR Fortessa flow cytometer (BD Biosciences).

### Statistics, reproducibility and analysis

Statistical analyses and graphics were carried out with Prism (GraphPad Software). Datasets were compared using different tests described in the legends. *P* values less than 0.05 were considered statistically significant. Data distribution was assumed to be normal, but this was not formally tested. No statistical method was used to predetermine sample size, but our sample sizes are similar to those calculated for similar experiments in previous publications^40,56^. Data collection and analysis of the human samples were performed blind to the conditions of the experiments. No animals or data points were excluded from the analyses except in Fig. 3c, where mice exhibiting an engraftment less than 1% hCD45^+^ cells were removed from the analysis because the hHSC number was too low to allow reliable quantification.

### Reporting summary

Further information on research design is available in the Nature Portfolio Reporting Summary linked to this article.

## Supplementary information

Reporting Summary

## Source data

Source Data Fig. 1 Statistical source data for Fig. 1.

Source Data Fig. 2 Statistical source data for Fig. 2.

Source Data Fig. 3 Statistical source data for Fig. 3.

Source Data Fig. 4 Statistical source data for Fig. 4.

Source Data Fig. 5 Statistical source data for Fig. 5.

Source Data Fig. 6 Statistical source data for Fig. 6.

Source Data Fig. 7 Statistical source data for Fig. 7.

Source Data Fig. 8 Statistical source data for Fig. 8.

Source Data Extended Data Fig. 1 Statistical source data for Extended Data Fig. 1.

Source Data Extended Data Fig. 2 Statistical source data for Extended Data Fig. 2.

Source Data Extended Data Fig. 3 Statistical source data for Extended Data Fig. 3.

Source Data Extended Data Fig. 4 Statistical source data for Extended Data Fig. 4.

Source Data Extended Data Fig. 5 Statistical source data for Extended Data Fig. 5.

Source Data Extended Data Fig. 6 Statistical source data for Extended Data Fig. 6.

Source Data Extended Data Fig. 7 Statistical source data for Extended Data Fig. 7.

Source Data Extended Data Fig. 8 Statistical source data for Extended Data Fig. 8.

Source Data Extended Data Fig. 9 Statistical source data for Extended Data Fig. 9.

Source Data Extended Data Fig. 10 Statistical source data for Extended Data Fig. 10.

## Data Availability

The data that support the findings of this study are included in the paper or available from the corresponding author upon reasonable request. Further information on research design is available in the Nature Research Reporting Summary linked to this article. Source data for Figs. 1–8 and Extended Data Figs. 1–10 have been provided as Source Data files. Source data are provided with this paper.

## Code availability

This study did not generate new code.
