# Supplementary material for: Different niches for stem cells carrying the same oncogenic driver affect pathogenesis and therapy response in myeloproliferative neoplasms
Source: Nat Cancer. 2023 Aug 7;4(8):1193–209. doi: 10.1038/s43018-023-00607-x (PMC10447237; doi:10.1038/s43018-023-00607-x)
Supplement: Supplementary file 1 — Reporting Summary [file 43018_2023_607_MOESM1_ESM.pdf]

nature portfolio

Corresponding author(s): Simon Mendez Ferrer

Last updated by author(s): May 5, 2023

Reporting Summary

Nature Portfolio wishes to improve the reproducibility of the work that we publish. This form provides structure for consistency and transparency in reporting. For further information on Nature Portfolio policies, see our Editorial Policies and the Editorial Policy Checklist.

Statistics

For all statistical analyses, confirm that the following items are present in the figure legend, table legend, main text, or Methods section.

n/a	Confirmed	
☐	☒	The exact sample size (*n*) for each experimental group/condition, given as a discrete number and unit of measurement
☐	☒	A statement on whether measurements were taken from distinct samples or whether the same sample was measured repeatedly
☐	☒	The statistical test(s) used AND whether they are one- or two-sided<br>*Only common tests should be described solely by name; describe more complex techniques in the Methods section.*
☐	☒	A description of all covariates tested
☐	☒	A description of any assumptions or corrections, such as tests of normality and adjustment for multiple comparisons
☐	☒	A full description of the statistical parameters including central tendency (e.g. means) or other basic estimates (e.g. regression coefficient) AND variation (e.g. standard deviation) or associated estimates of uncertainty (e.g. confidence intervals)
☐	☒	For null hypothesis testing, the test statistic (e.g. *F*, *t*, *r*) with confidence intervals, effect sizes, degrees of freedom and *P* value noted<br>*Give P values as exact values whenever suitable.*
☒	☐	For Bayesian analysis, information on the choice of priors and Markov chain Monte Carlo settings
☒	☐	For hierarchical and complex designs, identification of the appropriate level for tests and full reporting of outcomes
☒	☐	Estimates of effect sizes (e.g. Cohen's *d*, Pearson's *r*), indicating how they were calculated

Our web collection on statistics for biologists contains articles on many of the points above.

Software and code

Policy information about availability of computer code

Data collection	No open source or commercial code was used for the data collection in this manuscript.
Data analysis	Software used: Kaluza analysis 2.1, FlowJo v10.7.2, FACsDiva 6.0, CellProfiler 4.2.5, ImageJ (FIJI), Volocity 6.3, Imaris 9.2, NDP2 2.9.25, SkyScan CT 1272, GraphPad Prism 7, Biorender. CellProfiler (version 3.1.9) was used in figure 3 to analyze the number of cells from different populations in a defined area, in bone marrow sections. the pipeline can be found on GitHub : https://github.com/eg571/pipeline_CellProfiler_Grockowiak_et_al

For manuscripts utilizing custom algorithms or software that are central to the research but not yet described in published literature, software must be made available to editors and reviewers. We strongly encourage code deposition in a community repository (e.g. GitHub). See the Nature Portfolio guidelines for submitting code & software for further information.

Data

Policy information about availability of data

All manuscripts must include a data availability statement. This statement should provide the following information, where applicable:

- Accession codes, unique identifiers, or web links for publicly available datasets
- A description of any restrictions on data availability
- For clinical datasets or third party data, please ensure that the statement adheres to our policy

Data Availability statement: The data that support the findings of this study are included in the paper or available from the corresponding author upon reasonable request. Source data for Fig. 1-8 and Extended Data Fig. 1-10 have been provided as Source Data files.
We used a dataset published in the following publication : Tong, J., et al. Hematopoietic Stem Cell Heterogeneity Is Linked to the Initiation and Therapeutic Response of Myeloproliferative Neoplasms. Cell stem cell 28, 502-513 e506 (2021).

Human research participants

Policy information about studies involving human research participants and Sex and Gender in Research.

Reporting on sex and gender	We used some patients derived material (described in the methods), female and male MPN patients. Differences regarding the sex wasn't investigated in this study. The information on the disaggregation between sex and gender wasn't collected.
Population characteristics	The covariate characteristics that were considered were the disease type (Essential Thrombocythemia, or Polycythemia Vera), the moment when the sample was taken (before treatment or after 12months of therapy), and the therapy type (best available therapy or ruxolitinib).
Recruitment	In the MAJIC trial, MPN patients aged ≥18 with high-risk ET or PV, who met modified criteria for intolerance or resistance to HC were recruited, for more information please check : doi: 10.1182/blood-2017-05-785790. The Primary Thrombocythemia 1 (PT-1) study included newly diagnosed and previously treated MPN patients, 18 years of age or older, who met the Polycythemia Vera Study Group criteria for ET. Patients were recruited into 1 of 3 multicenter studies: the Medical Research Council high-risk trial, in which high-risk patients were randomly assigned to either hydroxyurea plus aspirin or to anagrelide plus aspirin; the National Cancer Research Institute intermediate-risk study, a randomization between aspirin alone or hydroxyurea plus aspirin; or the National Cancer Research Institute low-risk study, a prospective observational study of low-risk patients given aspirin alone. For more information please check : DOI: 10.1056/NEJMoa043800
Ethics oversight	All centres had appropriate research and ethical approval and patients gave their written informed consent.

Note that full information on the approval of the study protocol must also be provided in the manuscript.

Field-specific reporting

Please select the one below that is the best fit for your research. If you are not sure, read the appropriate sections before making your selection.

☒ Life sciences ☐ Behavioural & social sciences ☐ Ecological, evolutionary & environmental sciences

For a reference copy of the document with all sections, see nature.com/documents/nr-reporting-summary-flat.pdf

Life sciences study design

All studies must disclose on these points even when the disclosure is negative.

Sample size	The size of the mice cohorts were defined based on previous experience that the selected number of mice/samples should be sufficient to see an effect, taking into consideration the variability in the measurements, and the experience of our lab (Arranz et al. Nature 2014, Forte et al. Cell Metab. 2020). The sample size used in each experiment was not predetermined or formally justified for statistical power. Sample size for in vitro experiments were predetermined with sufficient replicates to validate experimental outcomes. For in vitro experiments, we didn't perform any statistical method to predetermine the sample size.
Data exclusions	No animals or data points were excluded from the analyses except in Figure3c, mice having a engraftment <1% hCD45+ cells were removed from the analysis because the hHSC number was too low to allow reliable quantification.
Replication	All attempts to replicate the experiments presented were successful. Some experiments presented in this paper were reproduced by different researchers at least twice for mice studies and sometimes only one experiment was shown to facilitate the presentation of the data. Experiments that couldn't be reproduced were not included from the paper. Every experiment performed in this study were a result of replication, the number of replicates is indicated in the legend of each figure.
Randomization	The allocation of mice to be injected with a specific type of cells in each experiment has been done randomly. Mice were allocated to control, or treatment group based on their blood count before the beginning of the treatment to have similar blood count in the different conditions.

Blinding	The experiments were performed blindly

Reporting for specific materials, systems and methods

We require information from authors about some types of materials, experimental systems and methods used in many studies. Here, indicate whether each material, system or method listed is relevant to your study. If you are not sure if a list item applies to your research, read the appropriate section before selecting a response.

Materials & experimental systems

n/a	Involved in the study
☐	☒ Antibodies
☒	☐ Eukaryotic cell lines
☒	☐ Palaeontology and archaeology
☐	☒ Animals and other organisms
☒	☐ Clinical data
☒	☐ Dual use research of concern

Methods

n/a	Involved in the study
☒	☐ ChIP-seq
☐	☒ Flow cytometry
☒	☐ MRI-based neuroimaging

Antibodies

Antibodies used	The following antibodies were used : goat anti-CD31 (R&D, AF3628 1:100), rat anti-EMCN (Insight Biotechnology, sc-65495 1:100), rabbit anti-Sp7 (Abcam, ab22552, 1:200), rat anti-CD31 (BD Biosciences, 550274, Clone MEC13.3; 1:200), goat-anti-OPN (R&D, AF808, 1:100), goat anti-CD117 (R&D, AF1356, 1:200), rabbit anti-Ki67 (Abcam, ab15580, 1:100), donkey anti-goat AF488 (Thermo Fisher, A11055), donkey anti-rabbit AF488 (Thermo Fisher, A21206), donkey anti-rat AF488 (Thermo Fisher, A21208), donkey anti-goat AF546 (Thermo Fisher, A11056), donkey anti-rabbit AF546 (Thermo Fisher, A10040), donkey anti-rat AF555 (Thermo Fisher, A21434), donkey anti-goat AF647 (Thermo Fisher, A21447), donkey anti-rabbit AF647 (Thermo Fisher, A31573), donkey anti-rat Dylight650 (Thermo Fisher SA5-10029), (rabbit-a-mouse CDC42, 1:100, Merck, cat. no. 07-1466), PE-conjugated mouse-anti-human CD45 (BD, cat. No. 555483), APC-conjugated mouse-anti-human CD34 (BD, cat. No. 555824), PE-Cy7-conjugated mouse-anti-human CD38 (BD, cat. No. 560677), biotin-conjugated lineage cocktail (BioLegend, cat. no. 133307), APC-Cy7-conjugated anti-Sca1 (BioLegend, cat. no. 108126), FITC-conjugated anti-CD117 (BioLegend, cat. no. 105805), BV711-conjugated anti-CD48 (BioLegend, cat. no. 103439), PE-Cy7-conjugated CD150 (BioLegend, cat. no. 115914), PE-conjugated streptavidin (BioLegend, cat. no. 405207), APC-Cy7-conjugated anti-CD117 (BioLegend, cat. no. 105826), BV421-conjugated anti-Sca1 (BioLegend, cat. no. 108128), BV605-conjugated anti-CD150 (BioLegend cat. no. 115927), PE-CY7-conjugated anti-CD117 (BioLegend, cat. no. 105814), APC-Cy7-conjugated anti-CD45.2 (Insight 25-0454-U100), BV605-conjugated anti-CD41 (BioLegend, cat. no. 133921), BV711-conjugated anti-CD16/CD32 (BioLegend cat. no. 101337), APC-conjugated anti-CD71 (Thermo Fisher, cat. no. 17-0711-80), BV605-conjugated anti-Ter119 (BioLegend, cat. no 116239), FITC-conjugated anti-CD34 (BD, cat. no. 553733), biotin-conjugated anti-CD3e (BD, cat. no BD 553060), PE-conjugated anti-Ly6G (BioLegend, cat. no 108408), BV421-conjugated anti-CD11b (BioLegend, cat. no 101235), BV510-conjugated streptavidin (BioLegend, cat. no. 405234), AF488-conjugated streptavidin (Invitrogen, cat. No. 405235), APC-conjugated anti-Ki67-antibody (BioLegend, cat. no. 652405), Hoechst 33342 (Thermo Fischer, cat. no. 62249), anti-STAT1 antibody (Thermo Fisher, cat. no PA5-95442), anti-STAT5 antibody (Cell Signalling, cat. no. 94205S), anti-Ter119 (BD, cat. no. 553672), biotin-conjugated anti-CD45 (BD, cat. no. 553078), PE-Cy7-conjugated anti-CD31 (BioLegend, cat. no. 102524), PE-conjugated anti-EMCN (Santa Cruz, cat. no. sc-665495 PE), APC-Cy7-conjugated anti-Sca1 (BioLegend, cat. no. 108126), BV421-conjugated rat-anti-mouse CD51 (BD cat. No. 740062), APC-conjugated anti-CD140a (BioLegend cat. no. 135908), BV710-conjugated streptavidin (BioLegend cat. no.405241), anti-pSTAT5 (BD, cat. no. 612599), or isotype control (BD, cat. no. 557783), anti-pSTAT1 (BD, cat. no. 612597).
Validation	Antibodies were all purchased and validated by the supplier. Validation statement for each antibody listed above can be found on the manufacturer's wabsite.

Animals and other research organisms

Policy information about studies involving animals; ARRIVE guidelines recommended for reporting animal research, and Sex and Gender in Research

Laboratory animals	Nes-gfp82, FVB/N-Adrb3tm1Lowl/J (JAX stock number 006402), B6.129S(Cg)-Stat1tm1Dlv/J (JAX stock number 012606), B6.129S6-Stat5btm1Mam Stat5atm2Mam/Mmjax (JAX stock number 032053), B6.FVB-Tg(Acta2-DsRed)1Rkl/J (JAX stock number 031159) (Jackson Laboratories), Vwf-TdTomato55, Vav-Cre;JAK2-V617F41, Mx1-Cre;JAK2-V617F41, JAK2:V617F45, Scl-tTA;JAK2-V617F43, MISTRG mice, CALRdel/+, and congenic B6.SJL-Ptprca Pepcb/BoyJ (CD45.1), CD45.2 C57BL/6 mice (Charles River Laboratories), were used in this study. Vav-Cre;JAK2-V617F mice and CALRdel/+ were used as ET-like model. Vav-Cre;JAK2-V617F mice express active Cre in fetal and adult HSCs. Mx1-Cre;JAK2-V617F41, JAK2R/R and Scl-tTA;JAK2-V617F mice were used as independent PV-like models. MISTRG mice were used for PDX studies. The age of the mice was indicated in the methods and legends of the figure when relevant.
Wild animals	This study didn't involve any wild animal.

Reporting on sex	Both males and females mice were used in this study, we didn't observe significant differences regarding the results one considering one sex or the other.
Field-collected samples	This study didn't involve samples collected from the field.
Ethics oversight	Mice were housed in specific pathogen free facilities. All experiments using mice followed protocols approved by the Animal Welfare Ethical Committee (AWERB), according to United Kingdom Home Office regulations (PPL P0242B783). Mice were maintained at the University Hospital Zurich animal facility in accordance with the Swiss Federal Veterinary office. Animal experiments were approved by the cantonal veterinary office of Zurich, Switzerland. Primary WT or MPN mice were sacrificed at 10-13w.o or 25w.o (as mentionned in the legend of the experiments). Recipient mice were transplanted at 8-13weeks old.

Note that full information on the approval of the study protocol must also be provided in the manuscript.

Flow Cytometry

Plots

Confirm that:

☒ The axis labels state the marker and fluorochrome used (e.g. CD4-FITC).

☒ The axis scales are clearly visible. Include numbers along axes only for bottom left plot of group (a 'group' is an analysis of identical markers).

☒ All plots are contour plots with outliers or pseudocolor plots.

☒ A numerical value for number of cells or percentage (with statistics) is provided.

Methodology

Sample preparation	Xenograft: Primary human HSPCs (CD34+) cells were purified from patient BM or PB using Ficoll density gradient centrifugation and magnetically isolated using the MACS CD34 MicroBead Kit. Patient CD34+ cells were cryopreserved, thawed in IMDM 50% FCS, and re-suspended in 25µl PBS for injection. MISTRG mice were irradiated and transplanted intra-femorally with 1.5-3x105 CD34+ hHSPCs. Histology of mouse bones: Femurs and tibia were harvested and put in PBS, 2% PFA overnight. Bones were washed, decalcified, embedded in OCT, and snap frozen. Sections or whole mount were obtained using a cryostat. Alternatively, bones were embedded in paraffin and 5µm-thick sections were stained. Immunofluorescence of sorted HSCs: BM cells were immunomagnetically depleted of hematopoietic lineage marker-expressing cells using biotin-conjugated lineage cocktail and magnetic streptavidin-conjugated beads, following the manufacturer’s recommendations. BM or PB cell suspension was depleted of red blood cells, incubated with fluorescent antibody conjugates, and analysed with a flow cytometer or sorted using FACSAria equipped with FACSDiva Software.
Instrument	All instruments used in this study are specified in the Methods : we used the LSRFortessa flow cytometer (BD Biosciences, Franklin Lakes, NJ) and the sorter (FACS Aria cell sorter, BD Bioscience) equiped with FACSDiva Software (BD Biosciences).
Software	Data were analysed using Kaluza software (Beckman Coulter).
Cell population abundance	- Sort or cKit+Sca1+CD48-CD150+ cells isolated post LIn depletion : 0.1 % - 0.5% depending on sample type
Gating strategy	- HSCs : HSCs are defined as LSK CD48-CD150+ and were first gated in the cells from FSC/SSC gate, then singlets, then live cells (DAPI-), then Lin negative, then Sca1+cKiT+, then CD150+CD48-. positive/negative threshold was defined using single stained control cells compared to unstained control.

☒ Tick this box to confirm that a figure exemplifying the gating strategy is provided in the Supplementary Information.
